# One-step templated synthesis of chiral organometallic salicyloxazoline complexes

**DOI:** 10.1186/s13065-019-0565-z

**Published:** 2019-04-04

**Authors:** Mei Luo, Jing Cheng Zhang, Hao Yin, Cheng Ming Wang, Susan Morris-Natschke, Kuo-Hsiung Lee

**Affiliations:** 1 College of Chemistry and Chemical Engineering, Hefei University of Technology, Hefei, 230009 China; 2 Hefei National Laboratory for Physical Sciences at the Microscale, University of Science and Technology of China, Hefei, 230026 China; 3 Natural Products Research Laboratories, UNC Eshelman, School of Pharmacy, University of North Carolina, Chapel Hill, NC 27599-7568 USA; 4 Chinese Medicine Research and Development Center, China Medical, University and Hospital, Taichung, 40447 Taiwan

**Keywords:** One-step method, Salicyloxazoline complexes, Metal salts, 2-Cyanophenol, d- and l-Amino alcohols

## Abstract

**Background:**

The general approach to the synthesis of metal complexes begins with ligand synthesis, followed by ligand reaction with metal salts to afford organometallic complexes. Our research group first reported a one-pot multicomponent synthesis of chiral oxazolinyl–zinc complexes, in the presence of a large amount of ZnCl_2_ (0.4–2.6 equiv.), with the yields of some products reaching 90%.

**Results:**

Our prior strategy was extended to use copper, cobalt, nickel, manganese, palladium or platinum salts as the third component. The one-step method used 1.0 equivalent of a metal salt, such as M(OAc)_2_·nH_2_O or MCl_2_·nH_2_O (M: Cu, Co, Ni, Pd or Pt, n = 1, 2 or 4), as a reagent to generate chiral salicyloxazoline complexes **1**–**8** in the reaction of 2-cyanophenol with different d- and l-amino alcohols.

**Conclusion:**

Complexes **1**–**8** were obtained using a one-pot method with a sequential strategy. The reaction outcome was demonstrated for three-component reactions between metal salts, amino alcohols and 2-hydroxybenzonitrile to afford organometallic complexes in good yields (65–95%).

**Electronic supplementary material:**

The online version of this article (10.1186/s13065-019-0565-z) contains supplementary material, which is available to authorized users.

## Introduction

Chiral oxazolinyl organometallic complexes are very important catalysts in organic chemistry [1–9]. Several organometallic complexes containing 2-(2′-hydroxyphenyl)oxazolines are reported in the literature [10–28]. These complexes exhibit good catalytic effects in asymmetric Baeyer–Villiger reactions [16–18], cyclopropanations [27, 28], and reductions of perchlorate with sulfides under mild conditions [29]. The general approach to the synthesis of metal complexes begins with ligand synthesis, followed by ligand reaction with metal salts to afford organometallic complexes [30]. Our research group first reported a one-pot multicomponent synthesis of chiral oxazolinyl–zinc complexes [31], in the presence of a large amount of ZnCl_2_ (0.4–2.6 equiv.), with the yields of some products reaching 90%. Herein, we report that chiral salicyloxazoline metal complexes can be produced using 1.0 equiv. of copper, cobalt, nickel, manganese, palladium and platinum salts as the third component. The structures were confirmed using X-ray crystallography.

## Results and discussion

**Scheme 1:**
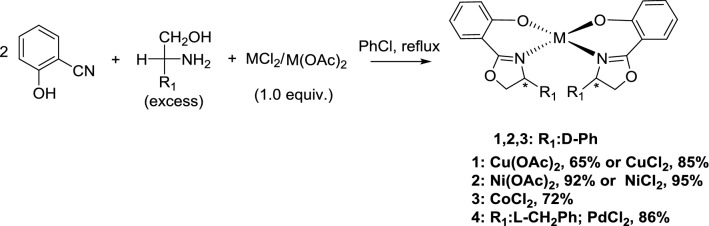
Templated synthesis of complexes **1**–**4**

Chiral bis(oxazoline) copper complex **1**, nickel complex **2**, cobalt complex **3** and palladium complex **4** were generated as crystals with the chemical formula ML_2_ (L = 2-(4-R_1_-4,5-dihydrooxazol-2-yl)phenol, R_1_: d-Ph, M: Cu, Ni, Co; R_1_: l-CH_2_Ph; M: Pd). The syntheses of these complexes are described below. A mixture of 2-hydroxybenzonitrile and d-phenylglycinol or l-phenylalaninol in 50 mL of chlorobenzene was refluxed for 72 h with 1.0 equiv. of the appropriate metal salt. After removal of chlorobenzene, purification was performed by recrystallization or column chromatography separation with petroleum ether and dichloromethane. Natural evaporation of the recrystallization or chromatographic solvent provided single crystals of chiral bisoxazolinyl metal complexes **1**–**4** (Scheme 1 and Additional file 1: Figures S1–S4).

**Scheme 2:**
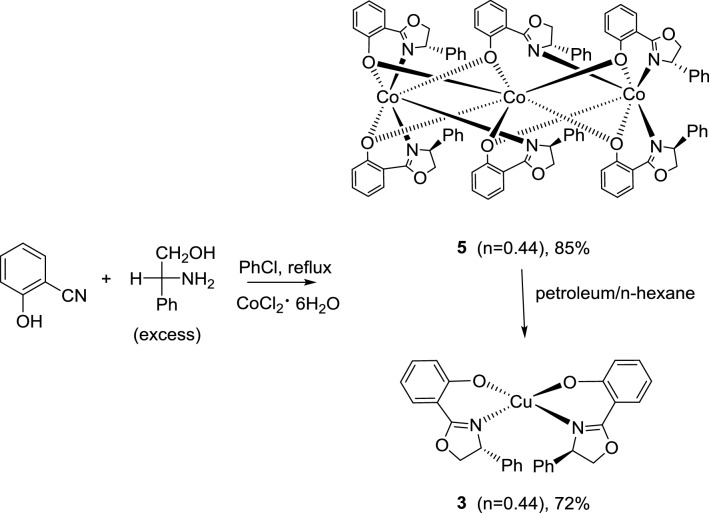
Effect of different solvents on the formation of complexes **3** and **5**

**Scheme 3:**
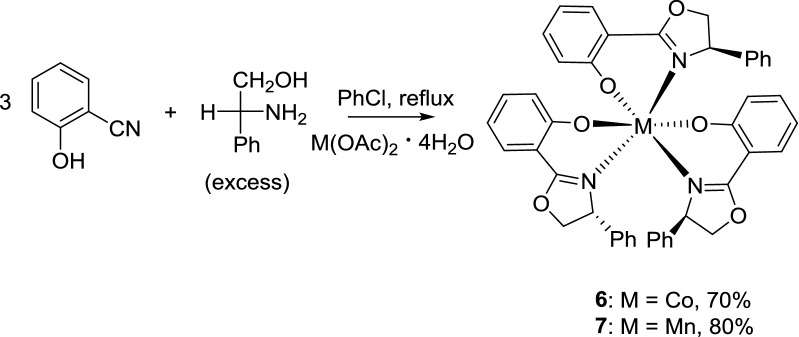
One-pot synthesis of tri(oxazoline) metal complexes **6** and **7**

**Fig. 1:**
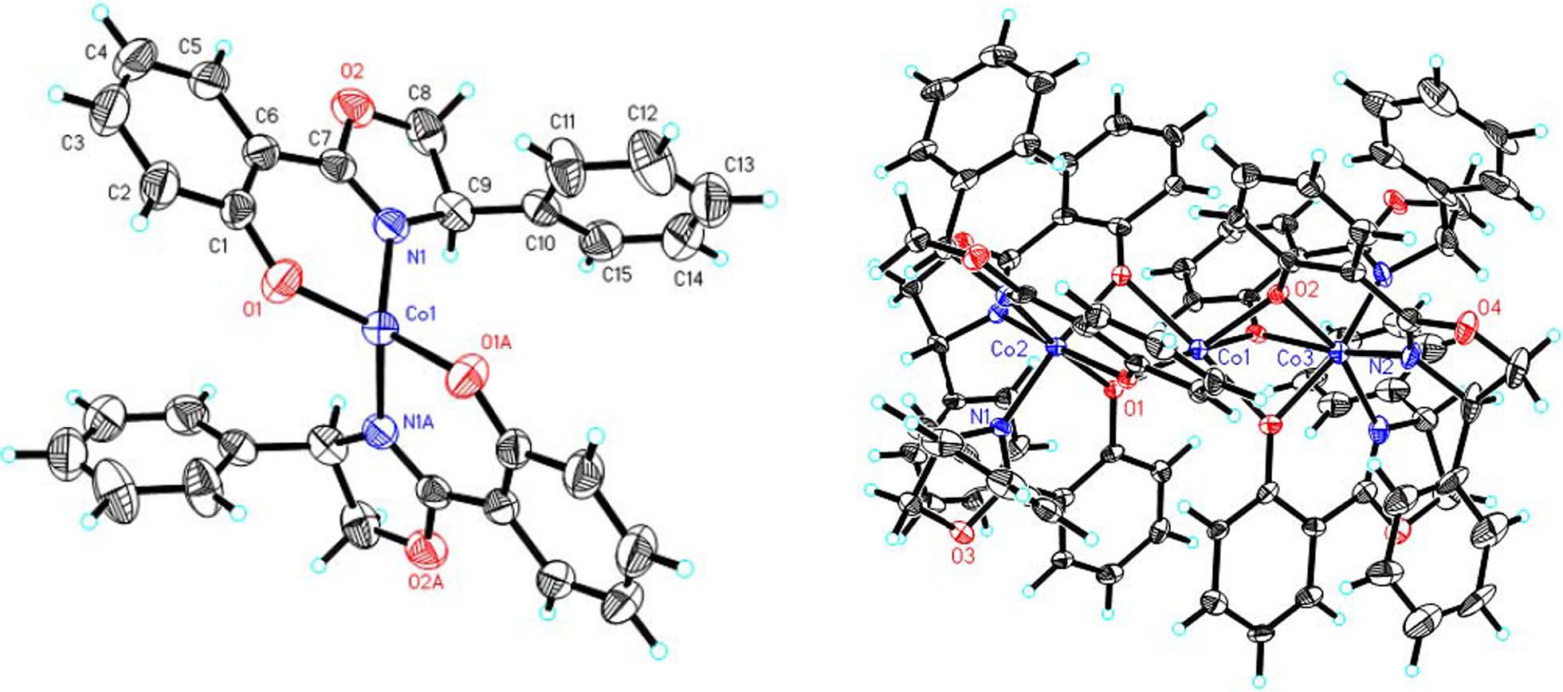
ORTEP view of complexes **3** (left) and **5** (right)

**Fig. 2:**
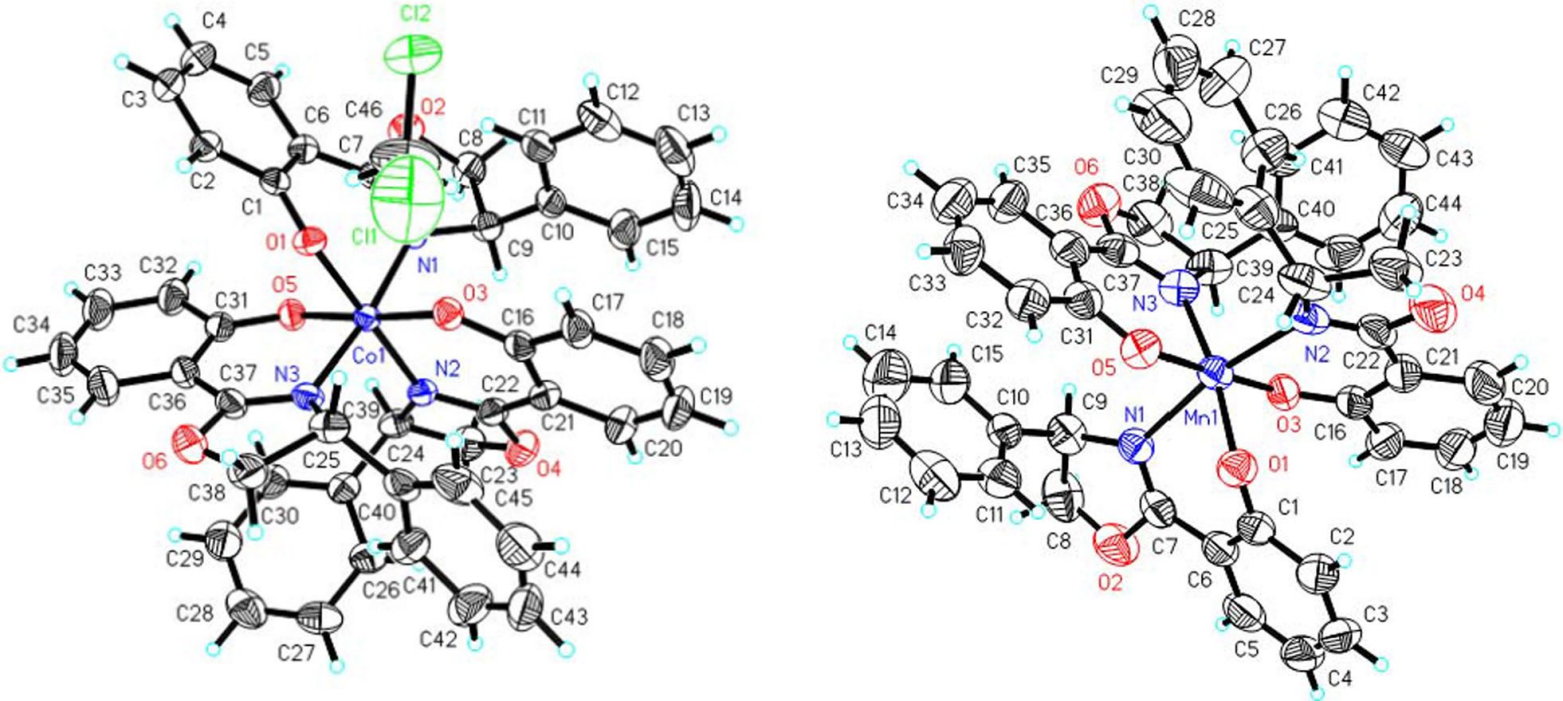
ORTEP view of complexes **6** (left) and **7** (right)

The chiral oxazoline cobalt complexes **5** and **6** were prepared by refluxing a mixture of 2-cyanophenol and d-phenylglycinol in chlorobenzene for 72 h with 1.0 equiv. of cobalt chloride hexahydrate or 1.0 equiv. of cobalt acetate tetrahydrate, respectively (Schemes 2 and 3, respectively). Crystals of complex **5** were obtained by slow evaporation from a 1:1 mixture of ethanol and chloroform (Fig. 1: right). However, the crystals of complex **6** were obtained after column chromatography with a 4:1 solution of petroleum ether and dichloromethane, followed by evaporation of the volatile components (Fig. 2: left).

Notably, the product complexes **3** and **5** were obtained using CoCl_2_ as a reagent with different solvents in the workup procedure. When a nonpolar solvent, such as petroleum ether or *n*-hexane, was used in the recrystallization medium, crystals of complex **3** were obtained. However, if the recrystallization was carried out with a mixture of two polar solvents, such as ethanol and chloroform, crystals of complex **5** were obtained (Scheme 2). Both crystal structures are shown in Fig. 1 (left: complex **3**, right: complex **5**).

Similarly, in the synthesis of chiral oxazoline manganese complex **7** by the title method, 2-hydroxybenzonitrile and d-phenylglycinol were dissolved in chlorobenzene and refluxed in the presence of 1.0 equiv. of manganese acetate tetrahydrate for 60 h (Scheme 3). Crystals of complex **7** (Fig. 2: right) were obtained by slow evaporation from a mixture of absolute ethanol and chloroform.

**Scheme 4:**
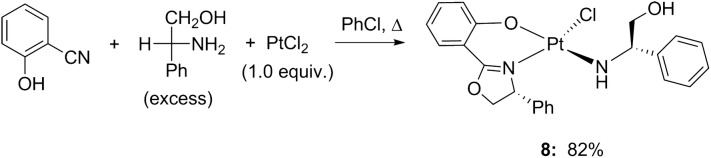
One-pot synthesis of oxazoline platinum complex **8**

**Fig. 3:**
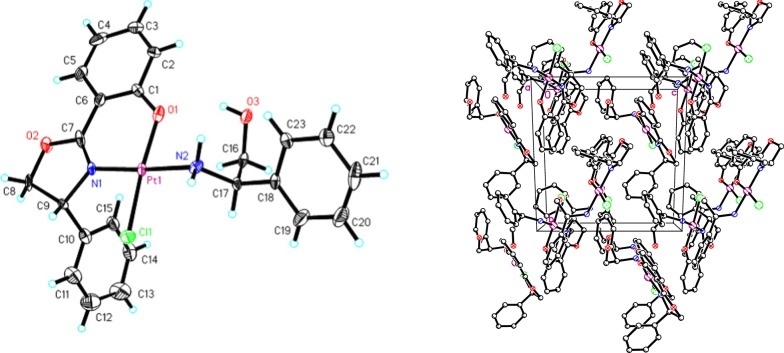
ORTEP view of complex **8** and packing of the molecule in a unit cell

Interestingly, when 1.0 equiv. of PtCl_2_ was employed in the reaction of 2-hydroxybenzonitrile with d-phenylglycinol in chlorobenzene, the crystal structure of the resulting Pt complex was different from those obtained with the previously mentioned metal salts. Complex **8**, which contains one unit of (*R*)-2-(4-phenyl-4,5-dihydrooxazol-2-yl)phenol and one unit of d-phenylglycinol, was obtained after column chromatography with petroleum ether and dichloromethane (4:1) followed by crystallization via slow evaporation (Scheme 4, Fig. 3).

The proposed mechanism indicates that the excess metal salts can activate the reaction of 2-hydroxybenzonitrile with d-phenylglycinol in chlorobenzene to form the ligand intermediates and then directly afford the corresponding organometallic complexes via a one-step procedure. Table 1 lists the summary of the metal salts used, the products obtained, and the percentage yields in the reactions.

**Table 1:** Summary of the metal salts used, the products obtained, and the percentage yields in the reactions

Metal salt	Amount of metal salt (mol%)	Products	Yield (%)
Cu(OAc)_2_·H_2_O	55.7	**1**	65
CuCl_2_·2H_2_O	53.2	**1**	85
Ni(OAc)_2_·4H_2_O	51.0	**2**	92
NiCl_2_· H_2_O	53.0	**2**	95
CoCl_2_·6H_2_O	44.3	**3**, **5**	72, 85
PdCl_2_	49.8	**4**	86
Co(OAc)_2_·4H_2_O	42.3	**6**	70
Mn(OAc)_2_·4H_2_O	52.6	**7**	80
PtCl_2_	33.7	**8**	82

In complexes **1**–**4**, the two oxazoline ligands arrange their donor atoms in a trans-planar configuration, and the structure features a four-coordinate metal center in a slightly distorted arrangement. The metal center is coordinated with the nitrogen atoms of the oxazolines and oxygen atom donated from the phenolate. The average length of the metal-N bond in complexes **1**–**4** are: Pd–N 2.003(7) Å > Co–N 1.983(5) Å > Cu–N 1.952(1) Å > Ni–N 1.893(3) Å, which are the same order as the average metal-O bond lengths for complexes **1**–**4** (e.g., Pd–O 1.986(6) Å > Co–O 1.925 (4) Å > Cu–O 1.924(9) Å > Ni–O 1.825(8) Å).

The crystal packing structure of complex **5** exhibits a sandwich-like structure and consists of three complex **3** (cobalt(II) chelates) connected by three Co(II) atoms, which generate 2D supramolecular networks. The molecular structure is depicted in Fig. 1 (right). The three cobalt (II) atoms in complex **5** form a linear trimer with a Co2–Co1–Co3 bond angle of 180°. In addition, the nonbonded Co···Co distances range from 2.823(3) to 2.832(3) Å, and the coordination sphere is different. The phenyl groups exhibit an all-cis arrangement. The central cobalt ion is at a highly symmetric center and coordinated to six hydroxyl oxygen atoms from the phenolates. The Co(1)–O bond lengths vary in the 2.059(5)–2.112(5) Å range, and the three equal bond lengths [2.059(5) Å] of Co(1)–O(2), Co(1)–O(2)#1 and Co(1)–O(2)#2 are shorter than the three equal bond lengths [2.112(5) Å] of Co(1)–O(1), Co(1)–O(1)#1 and Co(1)–O(1)#2. The two terminal cobalt (II) chelates Co(2) and Co(3) are octahedrally coordinated with two phenoxy ligands as well as one adjacent nitrogen atom and one pendant oxygen atom from separate Co(1) phenoxy ligands. The three equal distances for Co(2)–N are 1.923(5) Å and Co(3)–N are 2.098(6) Å, which are slightly longer than the corresponding Co–O bond lengths of 1.912(5) and 2.085(5) Å.

The molecular structures of complexes **6** and **7** were determined by single-crystal X-ray diffraction analysis. It is important to note that the entire molecule is in the independent part, occupying the general position of the P21/c symmetry group. In the structures of **6** and **7**, the O and N atoms from the three phenoxy ligands are coordinated to Co^3+^ or Mn^3+^ with distorted square planar geometries, and the three ligands lie in the adjacent positions. All coordinated ligands act as chelate-forming agents and close the rings using the metal cation. Due to the Jahn–Teller effect, the axial and equatorial Co–N bonds (1.956(2), 1.937(2) and 1.951(2) Å) in complex **6** are shorter than those of the Mn-N bonds in complex **7** (2.281(5), 2.024(5) and 2.024(5) Å). However, the Co–O bond lengths (1.895(18), 1.893(19) and 1.881(18) Å) are not the same order compared to the Mn–O bond lengths (1.930(4), 1.848(4), 1.870(4) Å).

The coordination angles for **6** and **7** vary between 84.2° and 179.9°. A slight compression of the M(phenox)_3_ units perpendicular to the Co: O(1)–N(3)–O(3), Mn: O(1)–O(3)–O(3) and the trigonal face was observed, leading to O(1)–Mn–N(1) angles of 81° and O(1)–Co(1)–N(3) and O(5)–Co(1)–N(1) angles of 84°. The crystal structure of complex **8** (Fig. 3, left) showed the presence of discrete mononuclear molecules, which were separated by van der Waals distances. The complex exhibits a nearly square-planar geometry with two equatorial nitrogen atoms, one from the oxazoline ring (Pt–N(1): 2.036 (14) Å) and one from the amino alcohol in the trans position (Pt–N(2): 1.999 (12) Å). The coordinated amine, hydroxyl group and uncoordinated hydroxyl group are involved in enriched supramolecular networks through hydrogen bonds (i.e., O(3)–H(3)···O(1) 2.813(18) Å and N(2)–H(2B)···O(3)#1 3.032(19)) (Fig. 3, right).

The contributions of the resonance structures shown in Additional file 1: Figures S1–S8 result in the C–N and C–O bonds of the oxazolyl ring exhibiting partial double bond character. A structure search of oxazolylphenolate complexes in the Cambridge Crystallographic Database resulted in an average C–N value of 1.291 Å (range 1.205–1.349 Å) and an average C–O value of 1.347 Å (range 1.304–1.424 Å). All C–N and C–O distances in the reported complexes fall within these expected ranges, and no systematic relationship was observed between the distance and the ligand torsion angle.

Some selected bond lengths and angles for all complexes are presented in Additional file 2: Table S1, and some hydrogen bond lengths and angles for complex **8** are also shown in Additional file 2: Table S2.

The X-ray crystal structures of the complexes were determined and are shown in the Additional file 1. In all cases, a distorted tetrahedral geometry is found at the metal(II) ion, and the C=N double bond character of the oxazoline ligand is largely retained in the metal complexes.

## Experimental

### General

Unless otherwise stated, 2-hydroxybenzonitrile, d-phenylglycinol, l-phenylalaninol, Cu(OAc)_2_·H_2_O, CuCl_2_·2H_2_O, Ni(OAc)_2_·H_2_O, NiCl_2_·6H_2_O, CoCl_2_·6H_2_O, Co(OAc)_2_·4H_2_O, Mn(OAc)_2_·4H_2_O, and PdCl_2_, PtCl_2_ were purchased from Acros, Aldrich, or Fluka (USA). Flash column chromatography was performed using Merck (Kenilworth, NJ, USA) silica gel (60, particle size 0.02–0.03 mm). The ^1^H and ^13^C NMR spectra were recorded using Bruker (Billerica, MA, USA) AM-500 or AM-600 spectrometers. The chemical shifts are reported in ppm (δ) with the solvent referenced to tetramethylsilane (TMS) as the internal standard (residual CHCl_3_, δ_H_ 7.26 ppm; CDCl_3_, δ_c_ 77 ppm). The following abbreviations were used to designate multiplicities: s = singlet, d = doublet, t = triplet, and m = multiplet. The infrared spectra were recorded on a Mattson Instruments (Madison, WI, USA) Galaxy Series FTIR 3000 spectrometer, and the peaks are reported in cm^−1^. Elemental analyses were obtained on an Elemental Analyzer AE-3000. The high-resolution mass spectra (HRMS) were obtained on a Micro GCT-MS (Waters, Rochester, MN, USA) equipped with an electron ionization (EI) ion source. Optical rotations were measured on a WZZ-1 automatic polarimeter with a 2 cm cell and recorded at the sodium d-line.

#### Bis(ligand) copper (II) chelate (CuL1_2_)

A dry 100 mL Schlenk flask was purged with N_2_ and charged with Cu(OAc)_2_·H_2_O (2.2198 g, 11.14 mmol) or CuCl_2_·2H_2_O (2.1199 g, 10.64 mmol), 2-cyanophenol (2.3808 g, 19.99 mmol) and d-phenylglycinol (3.8002–4.2003 g). Then, 40 mL of chlorobenzene was added, and the reaction mixture was refluxed for 72 h. After cooling to room temperature, the solvent was removed under reduced pressure, and the residue was dissolved in 15 mL of H_2_O followed by extraction with CH_2_Cl_2_ (3 × 20 mL). The combined organic extracts were evaporated to yield a crude green oil, which was purified by column chromatography (petroleum ether/CH_2_Cl_2_, 4/1) to afford the title compound as colorless crystals 1.9553 g in 65% yield or 2.4422 g in 85% yield; m.p.: > 240 °C, $\left[\upalpha \right]_{\text{D}}^{5}$ = + 235.7° (c = 0.0488, CH_3_OH). ν_max_ (cm^−1^): 3439, 3025, 2967, 2902, 1617, 1583, 1541, 1475, 1447, 1394, 1349, 1266, 1155, 1077, 1030, 949, 935, 855, 755, 695, 666, 574, 533, 414. Elemental analysis for C_30_H_24_N_2_O_4_Cu requires C: 66.72%, H: 4.44, N: 5.18%; found: C: 66.22%, H: 4.39%, N: 5.26%.

#### Bis(ligand) nickel (II) chelate (NiL1_2_)

Prepared using the procedure described for compound **1** by refluxing a mixture of 2-cyanophenol (2.3001 g, 19.33 mmol), Ni(OAc)_2_·4H_2_O (2.4528 g, 9.86 mmol) or NiCl_2_·6H_2_O (2.4374 g, 10.25 mmol) and d-phenylglycinol (4.2318 g) in 40 mL of dry chlorobenzene for 60 h. The product was obtained as dark brown crystals (2.5112 g in 92% yield or 2.6949 g) in 95% yield after column chromatography (petroleum ether/CH_2_Cl_2_, 4/1). m.p.: 196–198 °C, $\left[\upalpha \right]_{\text{D}}^{25}$ = + 119.57° (c = 0.0488, CH_3_OH), m.p.: 196–198 °C, $\left[\upalpha \right]_{\text{D}}^{25}$ =+ 119.57° (c = 0.0488, CH_3_OH), ^1^H NMR (600 MHz, CDCl_3_ and DMSO, 27°C): 7.85–7.86 (m, 2H), 7.22–7.49 (m, l2H), 6.46(d, J = 7.3 Hz, 2H), 6.30 (t, J = 6.4 Hz, 2H), 5.70–5.98 (m, 2H), 4.54–4.62 (m, 2H), 4.32–4.41 (m, 2H); δ_C_ (150 MHz, CDCl_3_): 164.5, 164.4, 142.3, 133.5, 127.3, 126.0, 125.7, 124.3, 113.1, 107.8, 107.7(× 2), 72.6, 72.5, 67.0, 65.1, 65.0. ν_max_ (cm^−1^): 3453, 3024, 2906, 1617, 1541, 1475, 1447, 1394, 1349, 1265, 1231, 1154, 1077, 1029,949, 931, 85,5, 755, 695, 574, 533, 415. Elemental analysis for C_30_H_24_N_2_O_4_Ni requires C: 67.32%, H: 4.52%, N: 5.23%; found: C: 67.22%, H: 4.39%, N: 5.26%.

#### Bis(ligand) cobalt (II) chelate (CoL1_2_)

Prepared using the procedure described for compound **1** by refluxing a mixture of CoCl_2_·6H_2_O (1.5671 g, 6.59 mmol), 2-cyanophenol (1.7699 g, 14.86 mmol) and d-phenylglycinol (3.6798 g) in 40 mL of dry chlorobenzene for 60 h. The product was obtained as red-brown crystals (1.7079 g) in 72% yield after evaporation from a mixture of petroleum or *n*-hexane, absolute ethanol or dichloromethane (1:1); m.p.: 146–147 °C, $\left[\upalpha \right]_{\text{D}}^{5}$ = − 149.2° (0.054, CH_3_OH), ^1^HNMR (600 MHz, CDCl_3_ and DMSO, 27 °C): 7.68 (d, J = 6.7 Hz, 2H), 7.29–7.48 (m, l4H), 6.95–7.00 (m, 2H), 5.50–5.53 (m, 2H), 4.87 (t, J = 8.4 Hz, 2H), 4.23–4.26 (m, 2H); δ_C_: 166.3, 160.0, 141.5, 133.6, 128.8, 128.2, 127.9 127.8, 126.5, 118.7, 116.8, 110.4, 74.0, 68.8. ν_max_ (cm^−1^): 3411, 3127, 2928, 1614,1617, 1591, 1536, 1493, 1475, 1456, 1439, 1388, 1255, 1236, 1157, 1074, 1056, 954, 932, 913, 851, 753, 699, 660, 615, 567, 530, 415. Elemental analysis for C_30_H_24_N_2_O_4_Co requires C: 67.54%, H: 4.53%, N: 6.34%; found: C: 67.87%, H: 4.86%, N: 6.33%.

#### Bis(ligand) palladium (II) chelate (PdL2_2_)

Prepared using the procedure described for compound **1** by refluxing a mixture of PdCl_2_ (0.8836 g, 4.98 mmol), 2-cyanophenol (1.1927 g, 10.01 mmol) and l-phenylalaninol (2.3579 g) in 40 mL of dry chlorobenzene for 60 h. The product was obtained as dark brown crystals (2.6166 g) in 86% yield after column chromatography (petroleum ether/CH_2_Cl_2_, 4/1); m.p.: 146–148 °C, $\left[\upalpha \right]_{\text{D}}^{5}$ = − 24.19° (c 0.0248, CH_3_OH): ^1^H NMR (300 MHz, CDCl_3_, 27°C), δ (ppm) = 7.44–7.47 (m, 5H), 7.24–7.31 (m, 10H), 6.85 (d, J = 0.8 Hz, 2H), 6.54 (t, J = 0.6 Hz, 1H), 4.74–4.78 (m, 2H), 4.49–4.54 (m, 3H), 3.35–3.41 (m, 2H), 2.83–2.91 (m, 3H); ^13^C NMR: 168.0, 162.3, 137.7, 134.7, 130.1, 129.9, 129.8, 129.2, 127.3, 121.7, 115.4, 109.5, 72.3, 62.3, 60.2, 54.5, 35.5. ν_max_ (cm^−1^): 3025, 1611, 1540, 1496, 1467, 1438, 1396, 1343 257, 1253, 1234, 1154, 1140, 1084, 1065, 1029, 973, 938, 856, 750, 725, 698, 683, 671, 620, 597, 577. Elemental analysis for C_32_H_28_N_2_O_4_Cl_2_Pd requires C: 56.36%, H: 4.14%, N: 4.11%; found: C: 56.48%, H: 4.22%, N: 4.38%.

#### Bis(ligand) cobalt (II) chelate trimer (CoL1_2_)_3_

Prepared using the procedure described for compound **1** by refluxing a mixture of CoCl_2_·6H_2_O (1.5671 g, 6.29 mmol), 2-cyanophenol (1.7699 g, 14.86 mmol) and d-phenylglycinol (3.8256 g) in 40 mL of dry chlorobenzene for 60 h. The product was obtained as red-brown crystals 6.0489 g in 85% yield after evaporation from a mixture of ethanol and chloroform (1:1); m.p.: 193–194 °C. ^1^H NMR (600 MHz, CDCl_3_ and DMSO, 27°C), δ (ppm) = 8.28 (d, J = 3.4 Hz, 1H), 7.71 (d, J = 7.2 Hz, 2H), 7.32–7.49 (m, 12H), 6.97–7.02 (m, 3H), 5.55 (d, J = 7.6 Hz, 2H), 4.91 (t, J = 9.0 Hz, 1H), 4.13–4.39 (m, 3H), δ_C_ (150 MHz, CDCl_3_, 27°C): 163.5, 157.3, 139.6, 131.9, 126.8, 126.7, 126.6, 126.0, 125.7, 125.6, 124.5, 117.0, 114.5, 108.0, 72.0, 65.9, 54.1, 46.7. ν_max_ (cm^−1^): 3061, 3030, 2965, 1613, 1550, 1478, 1441, 1397, 1339, 1232, 1157, 1073, 1057, 999, 952, 939, 856, 749, 698, 581. Elemental analysis for C_90_H_72_Co_3_N_6_O_12_ requires C: 67.30%, H: 4.48%, N: 5.23%; found: C: 67.23%, H: 4.70%, N: 4.90%.

#### Tri(ligand) cobalt chelate (CoL1_3_)

Prepared using the procedure described for compound **1** by refluxing a mixture of 1.5671 g of Co(OAc)_2_·4H_2_O (6.29 mmol), 2-cyanophenol (1.7699 g, 14.86 mmol) and d-phenylglycinol (3.6798 g) in 40 mL of dry chlorobenzene for 60 h. The product was obtained in 70% yield (2.5424 g) as dark brown crystals after column chromatography (petroleum ether/CH_2_Cl_2_, 4/1). Yield %: 70%; m.p.: 174–176 °C, $\left[\upalpha \right]_{\text{D}}^{5}$ = − 1014.1° (0.0212, CH_3_OH), δ_H_ (600 MHz, CDCl_3_, 27 °C) 7.50–7.52 (m, 1H), 7.23–7.24 (m, 1H), 7.02–7.07 (m, 2H), 6.87–6.97 (m, 9H), 6.74–6.80 (m, 7H), 6.56 (d, J = 8.56 Hz, 1H), 6.45–6.49 (m, 3H), 6.41 (d, J = 8.5 Hz, 1H), 6.24–6.27 (m, 2H), 5.45–5.48 (m, 1H), 5.29–5.32 (m, 1H), 4.91–4.92 (m, 2H), 4.79–4.82 (m, 2H), 4.33–4.36 (m, 1H), 4.26–4.28 (m, 2H); δ_C_ (150 MHz, CDCl_3_) 170.1, 170.0(× 2), 166.2, 165.3, 164.8, 140.3, 140.0(× 2), 133.1(× 2), 132.3, 128.1, 128.0, 127.7(× 4), 127.5, 127.4, 127.1, 126.8, 125.3, 124.4, 123.7, 123.0, 122.9, 113.9(× 2), 113.5, 113.1(× 2), 112.9, 109.2, 107.6, 76.3, 75.8, 75.2, 66.8, 66.1, 63.8. ν_max_ (cm^−1^): 3448, 3061, 1617, 1583, 1541, 1468, 1455, 1442, 1396, 1347, 1265, 1225, 1152, 1078, 949, 931, 856, 756, 747, 728, 696, 593, 577, 545, 409. Elemental analysis for C_46_H_38_Cl_2_N_3_O_6_Co requires C: 64.34%, H: 4.46, N: 4.89%; found: C: 64.48%, H: 4.27, N: 4.90%.

#### Tri(ligand) manganese chelate (MnL1_3_)

Prepared using the procedure described for compound **1** by refluxing a mixture of Mn(OAc)_2_·4H_2_O (2.5943 g, 10.59 mmol), 2-cyanophenol (2.3979 g, 20.13 mmol) and d-phenylglycinol (4.2681 g) in 40 mL of dry chlorobenzene for 60 h. The product was obtained as dark brown crystals (3.2390 g) in 80% yield after evaporation from a mixture of ethanol and chloroform; m.p.: 138–142 °C, $\left[\upalpha \right]_{\text{D}}^{5}$ = − 36.72° (c 0.488, CH_3_OH), δ_H_ (600 MHz, CDCl_3_, 27 °C) 7.75 (d, J = 1.4 Hz, 2H), 7.29–7.43 (m, 21H), 6.89–7.07 (m, 4H), 5.46–5.48 (m, 2H), 4.87–4.88 (m, 2H), 4.23–4.34 (m, 2H), 3.71–3.81 (m, 3H); δ_C_ (150 MHz, CDCl_3_), 165.8, 159.6, 141.1, 133.2, 128.4, 127.8, 127.4, 127.3, 126.6, 126.0, 118.3, 116.4, 110.0, 107.6, 73.6, 68.3; ν_max_ (cm^−1^): 3431, 3061, 3026, 2966, 2913, 1617, 1543, 1454, 1402, 1350, 1268, 1231, 1115, 1085, 939, 864. Elemental analysis for C_45_H_36_N_3_O_6_Mn requires C: 70.22%, H: 4.71%, N: 5.46%; found: C: 70.03%, H: 4.93%, N: 5.35%.

#### PtL1(d-phenylglycinol)Cl

Prepared using the procedure described for compound **1** by refluxing a mixture of dry PtCl_2_ (0.9026 g, 3.39 mmol), 2-cyanophenol (1.1959 g, 10.04 mmol) and d-phenylglycinol (4.3023 g). The three components were combined under water- and oxygen-free conditions in a dry 100 mL Schlenk flask. The components were dissolved in 80 mL of dry chlorobenzene, and the reaction mixture was refluxed for 60 h. The solvent was removed under reduced pressure, and the residue was dissolved in 15 mL of H_2_O followed by extraction with dichloromethane (10 × 3 mL). The solvent was removed under vacuum to afford the crude product as a red oil. Further purification was carried out using silica gel chromatography (petroleum ether/dichloromethane 4/1) to obtain the desired product as red-brown crystals (1.6718 g) in 82% yield; m.p.: 146–148 °C, $\left[\upalpha \right]_{\text{D}}^{5}$ = − 24.19° (c = 0.0248, CH_3_OH): ^1^H NMR (300 MHz, CDCl_3_, 27°C), δ (ppm) = 7.25–7.48 (m, 13H), 6.77–7.01 (m, 1H), 4.81–4.92 (m, 1H), 4.73–4.76 (m, 2H), 4.23–4.65 (m, 1H), 3.96–3.99 (m, 1H), 3.67–3.69 (m, 3H); δ_C_ (75 MHz, CDCl_3_): 159.9, 142.3, 140.2, 134.7, 129.5, 128.7, 128.6, 128.5, 128.4, 127.3, 119.8, 117.2, 74.6, 68.5, 64.9, 61.4. ν_max_ (cm^−1^): 3210, 3124, 2876, 2161, 2035, 1615, 1584, 1535, 1494, 1454, 1399, 1365, 1306, 1255, 1194, 1056, 1018, 910, 838, 754, 694, 619, 575, 566. Elemental analysis for C_23_H_22_N_2_O_3_ClPt requires C: 45.59%, H: 3.83, N: 4.62%; found: C: 45.16%, H: 4.22%, N: 4.86%.

## Conclusion

In conclusion, complexes **1**–**8** were obtained using a one-pot method with a sequential strategy [12]. The reaction outcome has been demonstrated for three-component reactions between metal salts, amino alcohols and 2-hydroxybenzonitrile to afford organometallic complexes in good yields (65–95%). Additionally, the dimeric complex **3** and the trimeric complex **5** can be obtained by selection of the appropriate polar and nonpolar solvents. Investigations of the catalytic properties of these complexes as chiral ligands are currently ongoing. These complexes have exhibited bioactivities as anticancer reagents, and their future use in medical fields are currently under development.

## Additional files

**Additional file 1.** Crystal structures of complexes **1**–**8**.

**Additional file 2.** Bond lengths, bond angles & crystal data for complexes **1**–**8**.

**Additional file 3.** NMR spectra of complexes **1**–**8**.
