# Correction to: One-step templated synthesis of chiral organometallic salicyloxazoline complexes

**DOI:** 10.1186/s13065-019-0577-8

**Published:** 2019-05-10

**Authors:** Mei Luo, Jing Cheng Zhang, Hao Yin, Cheng Ming Wang, Susan Morris-Natschke, Kuo-Hsiung Lee

**Affiliations:** 1 College of Chemistry and Chemical Engineering, Hefei University of Technology, Hefei, 230009 China; 2 Hefei National Laboratory for Physical Sciences at the Microscale, University of Science and Technology of China, Hefei, 230026 China; 3 Natural Products Research Laboratories, UNC Eshelman School of Pharmacy, University of North Carolina, Chapel Hill, NC 27599-7568 USA; 4 Chinese Medicine Research and Development Center, China Medical University and Hospital, Taichung, 40447 Taiwan

## Correction to: BMC Chem (2019) 13:51 10.1186/s13065-019-0565-z

Following publication of the original article [1], the authors reported an error in Schemes 1 and 2 and repeated line in subsection “Bis(ligand) nickel(II) chelate (NiL1_**2**_)”.

Please see below for the revised Schemes 1 and 2 and the corrected paragraph.

**Scheme 1:**
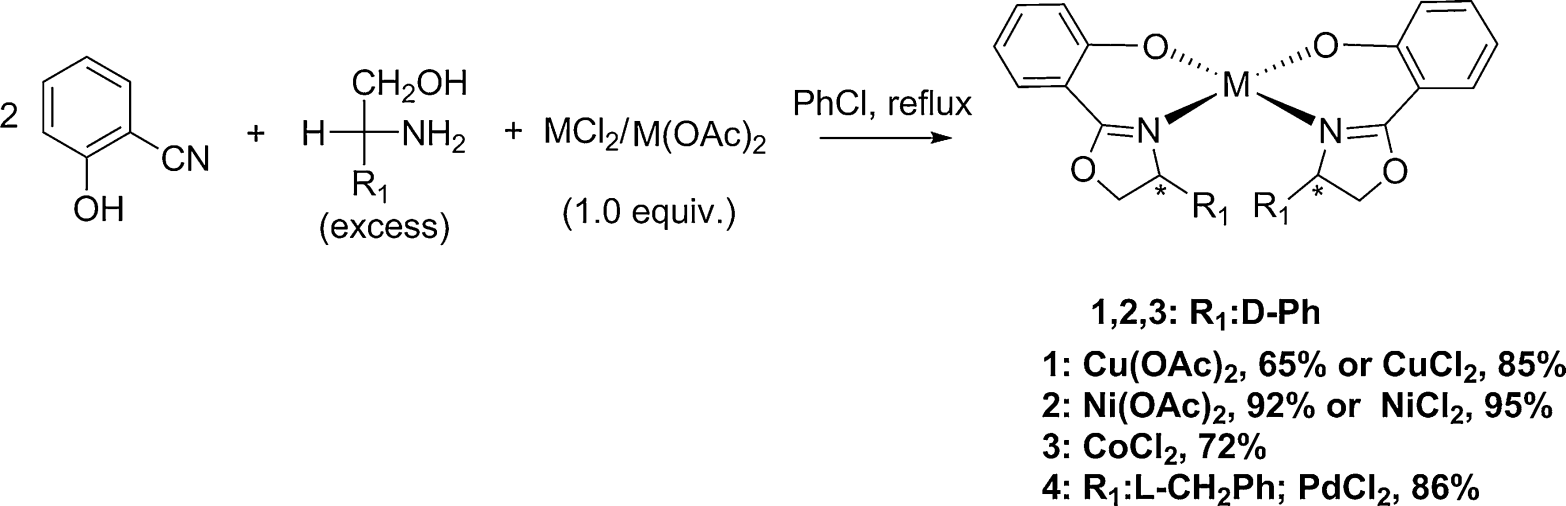
Templated synthesis of complexes **1**–**4**

**Scheme 2:**
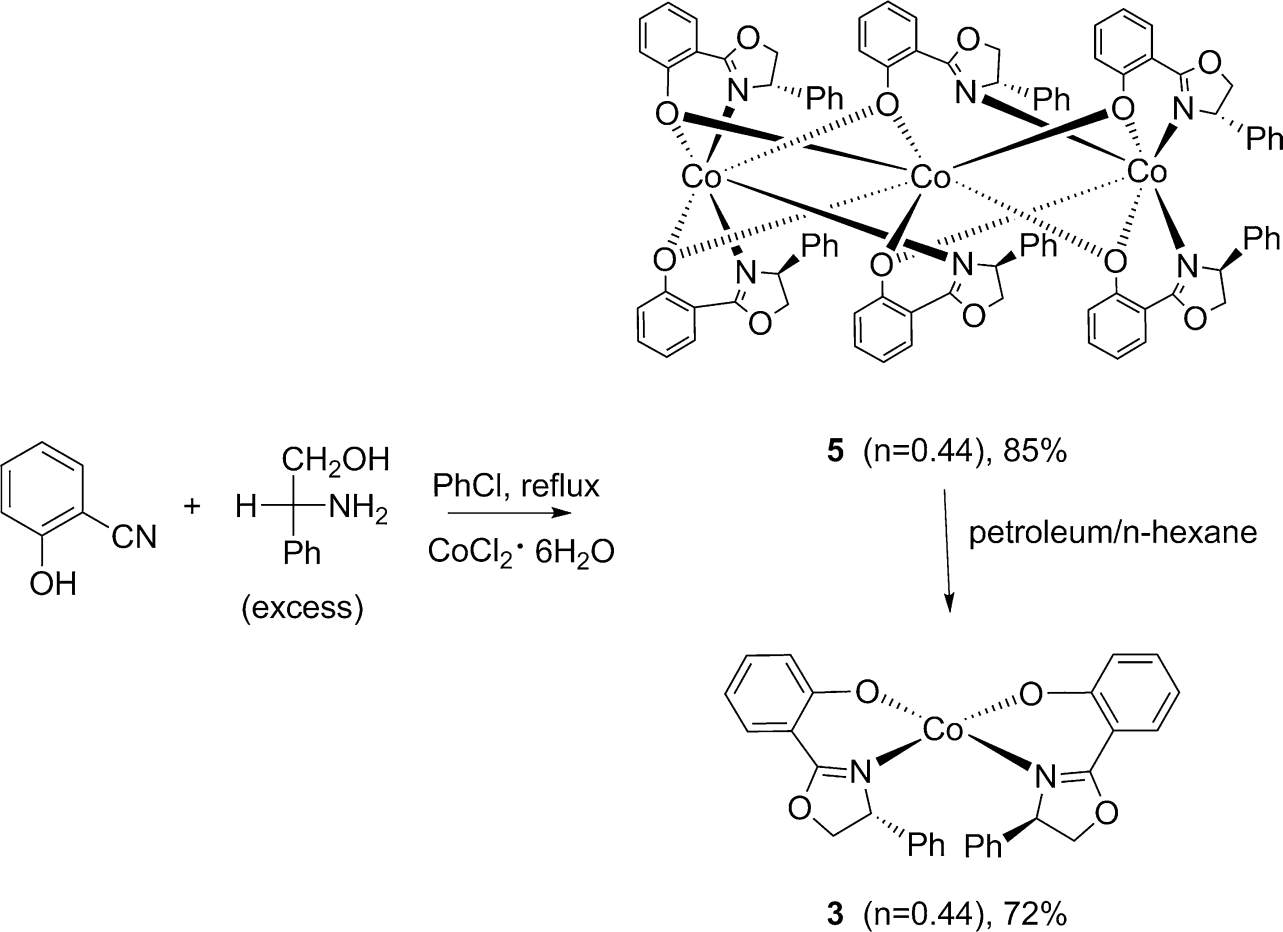
Effect of different solvents on the formation of complexes **3** and **5**

### Bis(ligand) nickel (II) chelate (NiL1_2_)

Prepared using the procedure described for compound **1** by refluxing a mixture of 2-cyanophenol (2.3001 g, 19.33 mmol), Ni(OAc)_2_·4H_2_O (2.4528 g, 9.86 mmol) or NiCl_2_·6H_2_O (2.4374 g, 10.25 mmol) and d-phenylglycinol (4.2318 g) in 40 mL of dry chlorobenzene for 60 h. The product was obtained as dark brown crystals (2.5112 g in 92% yield or 2.6949 g) in 95% yield after column chromatography (petroleum ether/CH_2_Cl_2_, 4/1). m.p.: 196–198 °C, $\left[\upalpha \right]_{\text{D}}^{25}$ = + 119.57° (c = 0.0488, CH_3_OH), ^1^H NMR (600 MHz, CDCl_3_ and DMSO, 27°C): 7.85–7.86 (m, 2H), 7.22–7.49 (m, l2H), 6.46(d, J = 7.3 Hz, 2H), 6.30 (t, J = 6.4 Hz, 2H), 5.70–5.98 (m, 2H), 4.54–4.62 (m, 2H), 4.32–4.41 (m, 2H); δ_C_ (150 MHz, CDCl_3_): 164.5, 164.4, 142.3, 133.5, 127.3, 126.0, 125.7, 124.3, 113.1, 107.8, 107.7(× 2), 72.6, 72.5, 67.0, 65.1, 65.0. ν_max_ (cm^−1^): 3453, 3024, 2906, 1617, 1541, 1475, 1447, 1394, 1349, 1265, 1231, 1154, 1077, 1029, 949, 931, 85,5, 755, 695, 574, 533, 415. Elemental analysis for C_30_H_24_N_2_O_4_Ni requires C: 67.32%, H: 4.52%, N: 5.23%; found: C: 67.22%, H: 4.39%, N: 5.26%.

### Tri(ligand) cobalt chelate (CoL1_3_)

Prepared using the procedure described for compound **1** by refluxing a mixture of 1.5671 g of Co(OAc)_2_·4H_2_O (6.29 mmol), 2-cyanophenol (1.7699 g, 14.86 mmol) and d-phenylglycinol (3.6798 g) in 40 mL of dry chlorobenzene for 60 h. The product was obtained in 70% yield (2.5424 g) as dark brown crystals after column chromatography (petroleum ether/CH_2_Cl_2_, 4/1). m.p.: 174–176 °C, [α]^5^_D_ = − 1014.1° (0.0212, CH_3_OH), δH(600 MHz, CDCl_3_, 27 °C) 7.50–7.52 (m, 1H), 7.23–7.24 (m, 1H), 7.02–7.07 (m, 2H), 6.87–6.97 (m, 9H), 6.74–6.80(m, 7H), 6.56 (d, J = 8.56 Hz, 1H), 6.45–6.49 (m, 3H), 6.41 (d, J = 8.5 Hz, 1H), 6.24–6.27 (m, 2H), 5.45–5.48 (m, 1H), 5.29–5.32 (m, 1H), 4.91–4.92 (m, 2H), 4.79–4.82 (m, 2H), 4.33–4.36 (m, 1H), 4.26–4.28 (m, 2H); δC (150 MHz, CDCl_3_) 170.1, 170.0, 168.9, 166.2, 165.3, 164.8, 140.3,139.8, 133.1(× 2), 132.3, 128.1, 128.0, 127.7,127.5, 127.4, 127.1, 126.8, 125.3, 124.4, 123.7, 122.9, 113.9, 113.5, 113.1), 112.9, 109.2, 107.6, 76.3, 75.8, 75.2, 66.8, 66.1, 63.8.ν_max_ (cm^−1^): 3448, 3061,1617, 1583, 1541, 1468, 1455, 1442, 1396, 1347, 1265,1225, 1152, 1078, 949, 931, 856, 756, 747, 728, 696, 593, 577, 545, 409. Elemental analysis for C_46_H_38_Cl_2_N_3_O_6_Co requires C: 64.34%, H: 4.46, N: 4.89%; found: C: 64.48%, H: 4.27, N: 4.90%.
