# Supplementary material for: One-step templated synthesis of chiral organometallic salicyloxazoline complexes
Source: BMC Chem. 2019 Apr 4;13(1):51. doi: 10.1186/s13065-019-0565-z (PMC6661745; doi:10.1186/s13065-019-0565-z)
Supplement: Supplementary file 1 — Additional file 1. Crystal structures of complexes 1–8. [file 13065_2019_565_MOESM1_ESM.doc]

**Crystal Structures of Complexes 1–8**

One-step templated synthesis of chiral organometallic salicyloxazoline complexes

**Contents:**

**Figures S1−S8.** Crystal Structures of Complexes **1**−**8** pages 2−4

Figure S1

**The crystal structure of complex 1**

**Figure S2**

**The crystal structure of complex 2**

**Figure S3**

**The crystal structure of complex 3**

**Figure S4**

**The crystal structure of complex 4**

**Figure S5**

**The crystal structure of complex 5**

**Figure S6**

**The crystal structure of complex 6**

**Figure S7**

**The crystal structure of complex 7**

**Figure S8**

**The crystal structure of complex 8**
