# Supplementary material for: One-step templated synthesis of chiral organometallic salicyloxazoline complexes
Source: BMC Chem. 2019 Apr 4;13(1):51. doi: 10.1186/s13065-019-0565-z (PMC6661745; doi:10.1186/s13065-019-0565-z)
Supplement: Supplementary file 2 — Additional file 2. Bond lengths, bond angles & crystal data for complexes 1–8. [file 13065_2019_565_MOESM2_ESM.doc]

**Bond Lengths, Bond Angles & Crystal Data for Complexes 1-8**

One-step templated synthesis of chiral organometallic salicyloxazoline complexes

**Contents:**

**Table S1.** Selected Bond Lengths (Å) and Bond Angles (º) for Complexes **1−8**  pages 2−3

**Table S2.** Hydrogen Bond Lengths (Å) and Bond Angles (°) for Complex **8** page 4

**Table S3.** Crystal Data and Structure Refinement for Complexes **1−8** pages 5−6

**Table S1.** Selected Bond Lengths (Å) and Bond Angles (º) for Complexes **1−8**

| **Bond (1)** | **Dist.** | **Bond (I)** | **Dist.** | **Bond (I)** | **Dist.** |
| --- | --- | --- | --- | --- | --- |
| Cu(1)-O(3) | 1.885(5) | Cu(1)-N(2) | 1.955(5) | Cu(2)-N(3) | 1.953(4) |
| Cu(1)-O(1) | 1.901(4) | Cu(2)-O(5) | 1.885(5) | Cu(2)-N(4) | 1.957(4 |
| Cu(1)-N(1) | 1.948(5) | Cu(2)-O(7) | 1.894(5) |  |  |
| **Angle (1)** | **(°)** | **Angle (I)** | **(°)** | **Angle (I)** | **(°)** |
| O(3)-Cu(1)-O(1) | 165.2(2) | O(1)-Cu(1)-N(2) | 89.24(19) | O(7)-Cu(2)-N(3) | 89.05(19) |
| O(3)-Cu(1)-N(1) | 90.4(2) | N(1)-Cu(1)-N(2) | 168.04(19) | O(5)-Cu(2)-N(4) | 88.62(18) |
| O(1)-Cu(1)-N(1) | 91.56(19) | O(5)-Cu(2)-O(7) | 175.3(2) | O(7)-Cu(2)-N(4) | 91.49(19) |
| O(3)-Cu(1)-N(2) | 91.8(2) | O(5)-Cu(2)-N(3) | 91.21(19) | N(3)-Cu(2)-N(4) | 175.4(2) |
| **Bond (2)** | **Dist** | **Bond (II)** | **Dist** | **Bond (II)** | **Dist** |
| Ni(1)-O(4) | 1.819(3) | Ni(1)-N(2) | 1.897(3) | Ni(2)-N(3) | 1.881(3) |
| Ni(1)-O(2) | 1.832(3) | Ni(2)-O(6) | 1.829(3) | Ni(2)-N(4) | 1.893(3) |
| Ni(1)-N(1) | 1.889(2) | Ni(2)-O(8) | 1.828(3) |  |  |
| **Angle (2)** | **(°)** | **Angle (I)** | **(°)** | **Angle (I)** | **(°)** |
| O(4)-Ni(1)-O(2) | 177.12(13) | O(2)-Ni(1)-N(2) | 87.71(11) | O(8)-Ni(2)-N(3) | 87.63(12) |
| O(4)-Ni(1)-N(1) | 87.02(11) | N(1)-Ni(1)-N(2) | 177.88(12) | O(6)-Ni(2)-N(4) | 87.71(11) |
| O(2)-Ni(1)-N(1) | 92.74(11) | O(6)-Ni(2)-O(8) | 172.11(13) | O(8)-Ni(2)-N(4) | 92.74(12) |
| O(4)-Ni(1)-N(2) | 92.63(11) | O(6)-Ni(2)-N(3) | 92.75(11) | N(3)-Ni(2)-N(4) | 173.97(12) |
| **Bond (3)** | **Dist** | **Bond (3)** | **Dist** | **Bond (3)** | **Dist** |
| Co(1)-O(1) | 1.925(4) | Co(1)-O(1)#1 | 1.925(4) | Co(1)-N(1)#1 | 1.983(5) |
| Co(1)-N(1) | 1.983(5) |  |  |  |  |
| **Angle (3)** | **(°)** | **Angle (4)** | **(°)** | **Angle (3)** | **(°)** |
| O(1)-Co(1)-O(1)#1 | 108.4(3) | O(1)#1-Co(1)-N(1)#1 | 93.19(18) | O(1)#1-Co(1)-N(1) | 126.89(18) |
| O(1)-Co(1)-N(1)#1 | 126.90(18) | O(1)-Co(1)-N(1) | 93.19(18) | N(1)#1-Co(1)-N(1) | 111.8(3) |
| **Bond (4)** | **Dist** | **Bond (4)** | **Dist** | **Bond (3)** | **Dist** |
| Pd(1)-O(2)#1 | 1.986(6) | Pd(1)-O(2) | 1.986(6) | Pd(1)-N(1)#1 | 2.003(7) |
| Pd(1)-N(1) | 2.003(7) |  |  |  |  |
| **Angle (4)** | **(°)** | **Angle (I)** | **(°)** | **Angle (4)** | **(°)** |
| O(2)#1-Pd(1)-O(2) | 178.0(7) | O(2)-Pd(1)-N(1) | 91.5(2) | O(2)-Pd(1)-N(1)#1 | 88.4(2) |
| O(2)#1-Pd(1)-N(1)#1 | 91.5(2) | N(1)#1-Pd(1)-N(1) | 178.1(8) | O(2)#1-Pd(1)-N(1) | 88.4(2) |
| **Bond (5)** | **Dist** | **Bond (5)** | **Dist** | **Bond (5)** | **Dist** |
| Co(1)-O(2)#1 | 2.059(5) | Co(1)-Co(3) | 2.832(3) | Co(3)-O(2)#1 | 2.085(5) |
| Co(1)-O(2)#2 | 2.059(5) | Co(2)-O(1)#2 | 1.912(5) | Co(3)-O(2)#2 | 2.085(5) |
| Co(1)-O(2) | 2.059(5) | Co(2)-O(1)#1 | 1.912(5) | Co(3)-O(2) | 2.085(5) |
| Co(1)-O(1) | 2.112(5) | Co(2)-O(1) | 1.913(5) | Co(3)-N(2) | 2.098(6) |
| Co(1)-O(1)#2 | 2.112(5) | Co(2)-N(1)#2 | 1.923(5) | Co(3)-N(2)#2 | 2.098(6) |
| Co(1)-O(1)#1 | 2.112(5) | Co(2)-N(1) | 1.923(5) | Co(3)-N(2)#1 | 2.098(6) |
| Co(1)-Co(2) | 2.823(3) | Co(2)-N(1)#1 | 1.923(5) |  |  |
| **Angle (5)** | **(°)** | **Angle (5)** | **(°)** | **Angle (5)** | **(°)** |
| O(2)-Co(1)-O(1) | 170.99(19) | O(1)-Co(2)-Co(1) | 48.43(14) | C(24)-N(2)-Co(3) | 124.0(5) |
| O(2)-Co(1)-Co(2) | 132.71(14) | O(2)-Co(3)-N(2) | 85.4(2) | C(1)-O(1)-Co(2) | 128.3(4) |
| O(1)-Co(1)-Co(2) | 42.64(13) | O(2)-Co(3)-Co(1) | 46.52(14) | C(1)-O(1)-Co(1) | 121.4(4) |
| O(2)-Co(1)-Co(3) | 47.29(14) | N(2)-Co(3)-Co(1) | 119.80(17) | Co(2)-O(1)-Co(1) | 88.93(18) |
| O(1)-Co(1)-Co(3) | 137.36(13) | C(7)-N(1)-Co(2) | 123.6(5) | C(16)-O(2)-Co(1) | 132.7(4) |
| Co(2)-Co(1)-Co(3) | 180.0 | C(9)-N(1)-Co(2) | 128.6(4) | C(16)-O(2)-Co(3) | 132.9(4) |
| O(1)-Co(2)-N(1) | 93.5(2) | C(22)-N(2)-Co(3) | 127.2(5) | Co(1)-O(2)-Co(3) | 86.19(18) |
| **Bond (6)** | **Dist** | **Bond (6)** | **Dist** | **Bond (6)** | **Dist** |
| Co(1)-O(5) | 1.8814(18) | Co(1)-O(1) | 1.8946(18) | Co(1)-N(3) | 1.951(2) |
| Co(1)-O(3) | 1.8934(19) | Co(1)-N(2) | 1.937(2) | Co(1)-N(1) | 1.956(2) |
| **Angle (6)** | **(°)** | **Angle (6)** | **(°)** | **Angle (6)** | **(°)** |
| O(5)-Co(1)-O(3) | 176.44(9) | O(1)-Co(1)-N(3) | 84.26(9) | C(9)-N(1)-Co(1) | 132.15(19) |
| O(5)-Co(1)-O(1) | 90.34(9) | N(2)-Co(1)-N(3) | 95.67(10) | C(22)-N(2)-C(24) | 108.7(3) |
| O(3)-Co(1)-O(1) | 87.64(9) | O(5)-Co(1)-N(1) | 84.54(9) | C(22)-N(2)-Co(1) | 124.9(2) |
| O(5)-Co(1)-N(2) | 89.73(9) | O(3)-Co(1)-N(1) | 92.50(9) | C(24)-N(2)-Co(1) | 125.64(18) |
| O(3)-Co(1)-N(2) | 92.30(9) | O(1)-Co(1)-N(1) | 89.33(9) | C(37)-N(3)-Co(1) | 121.3(2) |
| O(1)-Co(1)-N(2) | 179.91(11) | N(2)-Co(1)-N(1) | 90.74(10) | C(39)-N(3)-Co(1) | 124.16(19) |
| O(5)-Co(1)-N(3) | 92.34(10) | N(3)-Co(1)-N(1) | 172.86(10) | C(1)-O(1)-Co(1) | 122.29(17) |
| O(3)-Co(1)-N(3) | 90.37(9) | C(7)-N(1)-Co(1) | 120.31(18) | C(16)-O(3)-Co(1) | 128.7(2) |
| C(31)-O(5)-Co(1) | 126.22(19) |  |  |  |  |
| **Bond (7)** | **Dist** | **Bond (7)** | **Dist** | **Bond (7)** | **Dist** |
| Mn(1)-O(3) | 1.848(4) | Mn(1)-O(1) | 1.930(4) | Mn(1)-N(2) | 2.204(5) |
| Mn(1)-O(5) | 1.870(4) | Mn(1)-N(3) | 2.024(5) | Mn(1)-N(1) | 2.281(5) |
| **Angle (7)** | **(°)** | **Angle (7)** | **(°)** | **Angle (7)** | **(°)** |
| O(3)-Mn(1)-O(5) | 178.02(19) | O(1)-Mn(1)-N(2) | 89.3(2) | C(9)-N(1)-Mn(1) | 134.2(4) |
| O(3)-Mn(1)-O(1) | 91.70(18) | N(3)-Mn(1)-N(2) | 100.7(2) | C(22)-N(2)-Mn(1) | 120.7(5) |
| O(5)-Mn(1)-O(1) | 90.06(19) | O(3)-Mn(1)-N(1) | 87.77(18) | C(24)-N(2)-Mn(1) | 127.2(5) |
| O(3)-Mn(1)-N(3) | 89.58(19) | O(5)-Mn(1)-N(1) | 93.39(18) | C(37)-N(3)-Mn(1) | 124.9(4) |
| O(5)-Mn(1)-N(3) | 88.8(2) | O(1)-Mn(1)-N(1) | 81.53(18) | C(39)-N(3)-Mn(1) | 124.8(4) |
| O(1)-Mn(1)-N(3) | 169.96(19) | N(3)-Mn(1)-N(1) | 88.57(18) | C(1)-O(1)-Mn(1) | 123.5(4) |
| O(3)-Mn(1)-N(2) | 87.5(2) | N(2)-Mn(1)-N(1) | 169.5(2) | C(16)-O(3)-Mn(1) | 131.9(4) |
| O(5)-Mn(1)-N(2) | 91.6(2) | C(7)-N(1)-Mn(1) | 116.8(4) | C(31)-O(5)-Mn(1) | 132.8(4) |
| **Bond (8)** | **Dist** | **Bond (8)** | **Dist** | **Bond (8)** | **Dist** |
| Pt(1)-Cl(1) | 2.291(4) | Pt(1)-O(1) | 1.967(14) | Pt(1)-N(2) | 2.036(14) |
| Pt(1)-N(1) | 1.999(12) |  |  |  |  |
| Angle (**8**) | (**°**) | Angle (**8**) | (**°**) | Angle (**8**) | (**°**) |
| O(1)-Pt(1)-Cl(1) | 173.9(4) | N(1)-Pt(1)-Cl(1) | 94.9(4) | C(7)-N(1)-Pt(1) | 125.7(11) |
| O(1)-Pt(1)-N(2) | 86.0(6) | N(1)-Pt(1)-N(2) | 176.9(6) | C(7)-N(1)-C(9) | 109.5(13) |
| O(1)-Pt(1)-N(1) | 91.1(5) | C(1)-O(1)-Pt(1) | 128.0(11) | C(9)-N(1)-Pt(1) | 124.8(9) |
| N(2)-Pt(1)-Cl(1) | 87.9(4) | C(17)-N(2)-Pt(1) | 118.2(9) |  |  |

**Table S2.** Hydrogen Bond Lengths (Å) and Bond Angles (°) for Complex **8**

| **D–H···A (complex 8)** | **d(D–H)** | **d(H···A)** | **d(D···A)** | **∠DHA** |
| --- | --- | --- | --- | --- |
| O3-H3…O1 | 0.820 | 2.109 | 2.813 | 143.86 |
| N2-H2B…O3#3 | 0.970 | 2.109 | 3.031 | 158.17 |

Symmetry codes: #3 -y+2, x-y+1, z+1/3

**Table S3.** Crystal Data and Structure Refinement for Complexes **1−8**.

| Complexes | 1 | 2 | 3 | 4 | 5 | 6 | 7 | 8 |
| --- | --- | --- | --- | --- | --- | --- | --- | --- |
| Empirical formula | C_50_H_42_F_6_N_4_PdS_2_O_11_ | C_30_H_24_NiN_2_O_4_ | C_16_H_26_Cl_2_CoN_6_O_4_ | C_32_H_28_Cl_2_PdN_2_O_4_ | C_90_H_72_Co_3_N_6_O_12_ | C_46_H_38_Cl_2_CoN_3_O_6_ | C_45_H_38_MnN_3_O_6_ | C_23_H_23_ClPtN_2_O_32_ |
| Formula weight | 1159.39 | 535.22 | 535.44 | 610.96 | 1606.33 | 858.62 | 769.71 | 605.97 |
| Temperature | 133(2)K | 293(2) K | 293(2)K | 273(2) K | 140(2) K | 293(2) K | 293(2) K | 296(2) K |
| Crystal system | Monoclinic | Monoclinic | Monoclinic | Orthorhombic | Rhombohedral | Orthorhombic | Orthorhombic | Trigonal |
| Space group | P 2(1) | P 2(1) | C 2 | C 2/221 | R3 | P2(1)2(1)2/(1) | P2(1)2(1)2/(1) | P3(1) |
| a / Å | 10.7419(11) | 9.8976(8) | 19.403(4) | 10.185(7) | 19.092(3) | 10.4118(6) | 10..2120(6) | 13.1105(3) |
| b / Å | 14.7446(16) | 23.0751(18) | 5.6909(11) | 18.769(16) | 19.092(3) | 11.3478(7) | 11.1056(14) | 13.1105(3) |
| c / Å | 16.1431(17) | 11.1087(9) | 11.562(2) | 14.196(10) | 18.575(5) | 33.833(2) | 35.702(5) | 11.1292(3) |
| β / ° | 107.1431(2) | 99.794(2) | 94.438(7) | 90 | 90 | 90 | 90 | 90 |
| γ/ ° | 90 | 90 | 90 | 120j | 120 | 90 | 90 | 120 |
| V / Å3 | 2443.2(4) | 2500.1(3) | 1272.9(4) | 2714(4) | 5864(2) | 3997.4(4) | 4049.0(9) | 1656.66(9) |
| Z | 4 | 4 | 2 | 4 | 3 | 4 | 4 | 3 |
| Dc / g.cm-3 | 1.576 | 1.422 | 1.397 | 1.495 | 1.365 | 1.427 | 1.263 | 1.822 |
| μ / mm-1 | 0.554 | 0.816 | 0.713 | 0.724 | 0.697 | 0.618 | 0.377 | 13.216 |
| F(000) | 1180 | 1112 | 554 | 1248 | 2493 | 1776 | 1600 | 882 |
| Completeness  to θ | 100.0 % | 99.9% | 99.9 % | 99.9 % | 100.0 % | 99.9 % | 99.7 % | 99.1 % |
| Crystal size (mm) | 0.211 x 0.165  x 0.121 | 0.187x0.165x0.112 | 0.160x0.110x0.050 | 0.178x0.132x 0.074 | 0.09x0.08x  0.06 | 0.254x0.175x  0.123 | 0.211x0.175x  0.121 | 0.20x0.15x  0.12 |
| Final R indices [I>2sigma(I)] | 0.0855 | 0.0361 | 0.0547 | 0.0480 | 0.0646 | 0.0430 | 0.0784 | 0.0644 |
| R indices (all data) | 0.1244 | 0.0436 | 0.0606 | 0.0590 | 0.1299 | 0.0526 | 0.1414 | 0.0645 |
| Largest diff. peak and hole | 2.646, -1.818 | .415, -0.207 | 0.575, -0.265 | 1.52, -0.519 | 0.632, -0.306 | 0.379, -0.323 | 0.482, -0.355 | 2.415, -4.821 |
