# Supplementary material for: One-step templated synthesis of chiral organometallic salicyloxazoline complexes
Source: BMC Chem. 2019 Apr 4;13(1):51. doi: 10.1186/s13065-019-0565-z (PMC6661745; doi:10.1186/s13065-019-0565-z)

**NMR Spectra of Complexes 1–8**

One-step templated synthesis of chiral organometallic salicyloxazoline complexes

**Contents:**

Copies of NMR Spectra pages 2−8

**Complex 2**


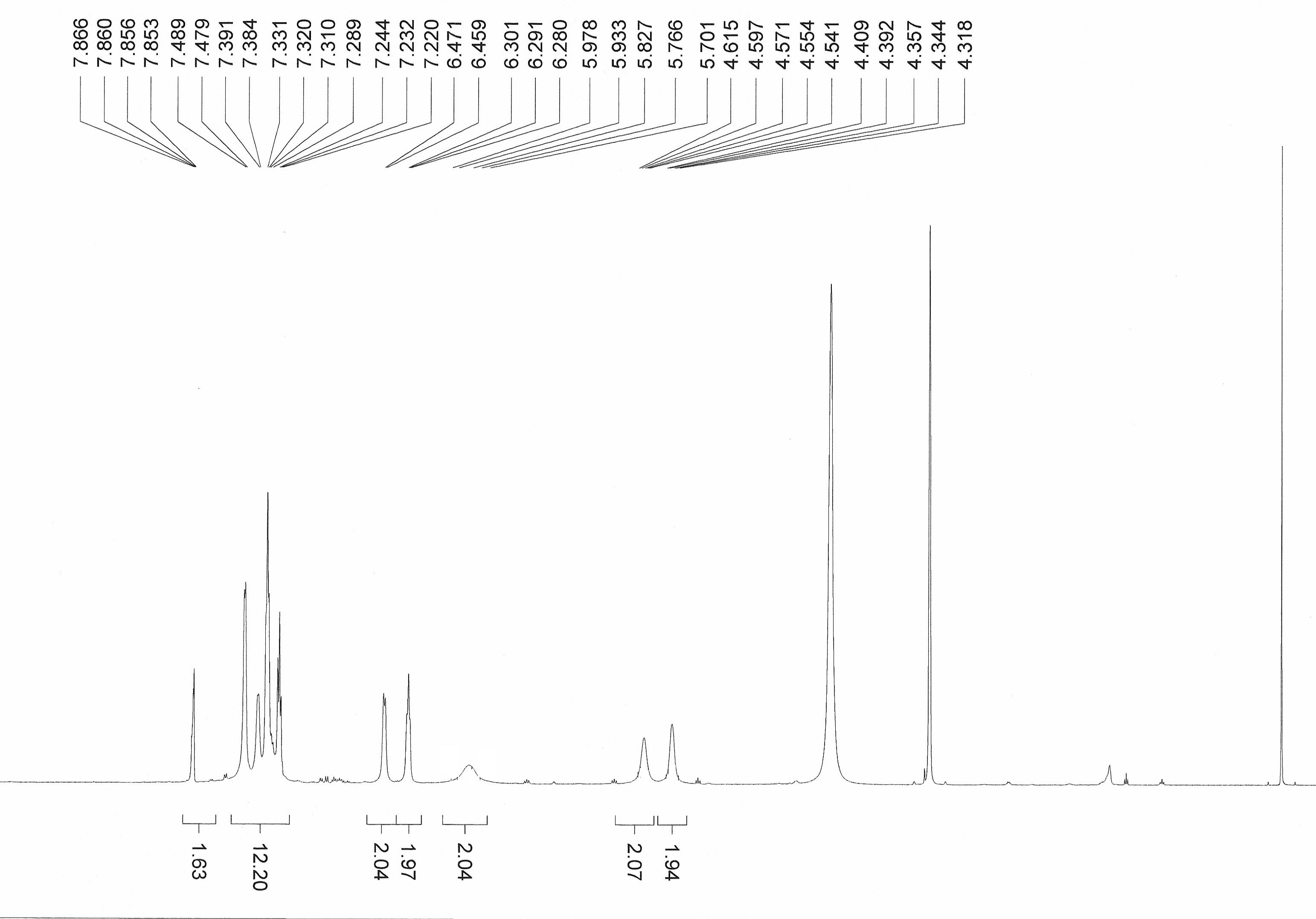


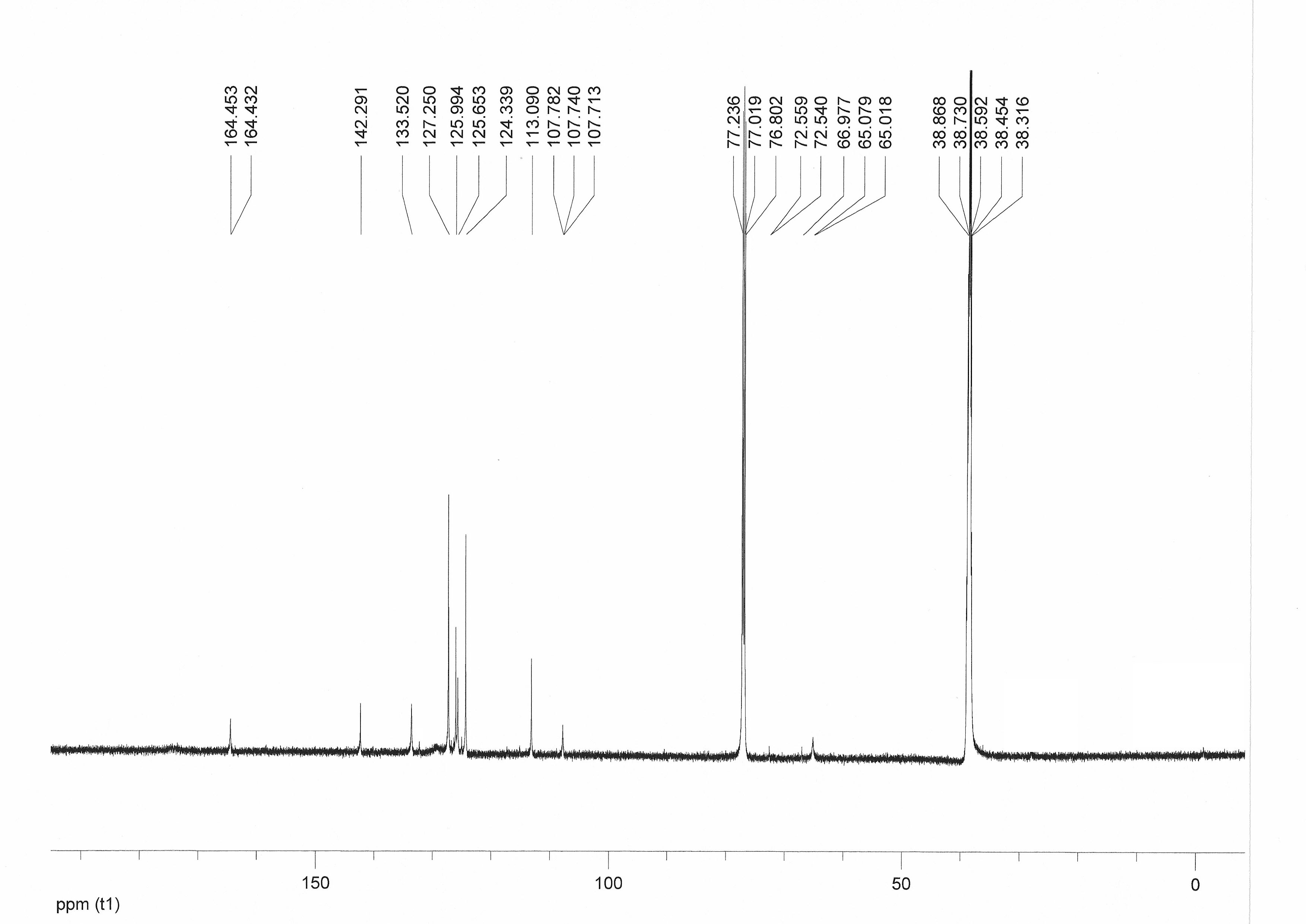


**Complex 3**

**
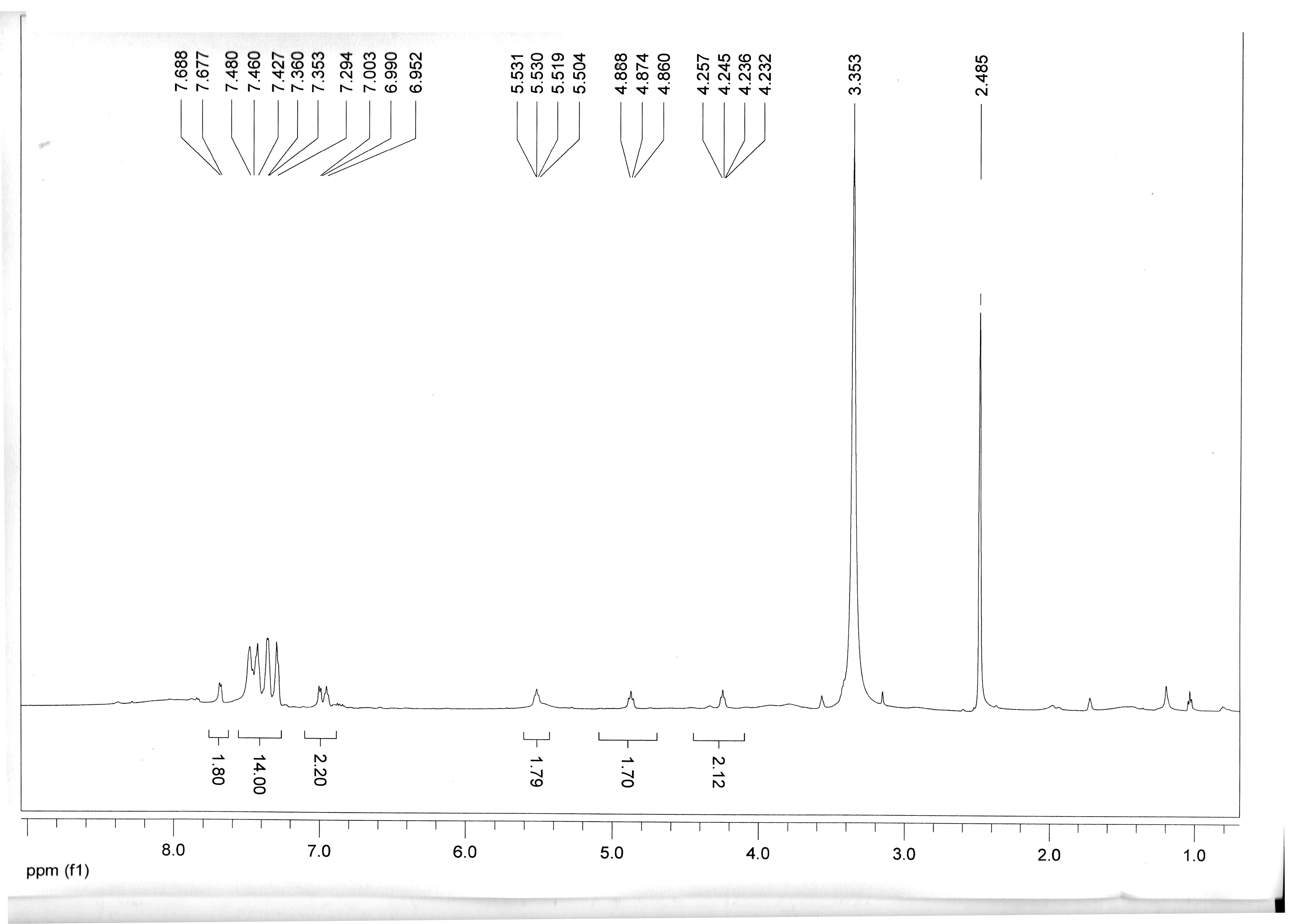
**


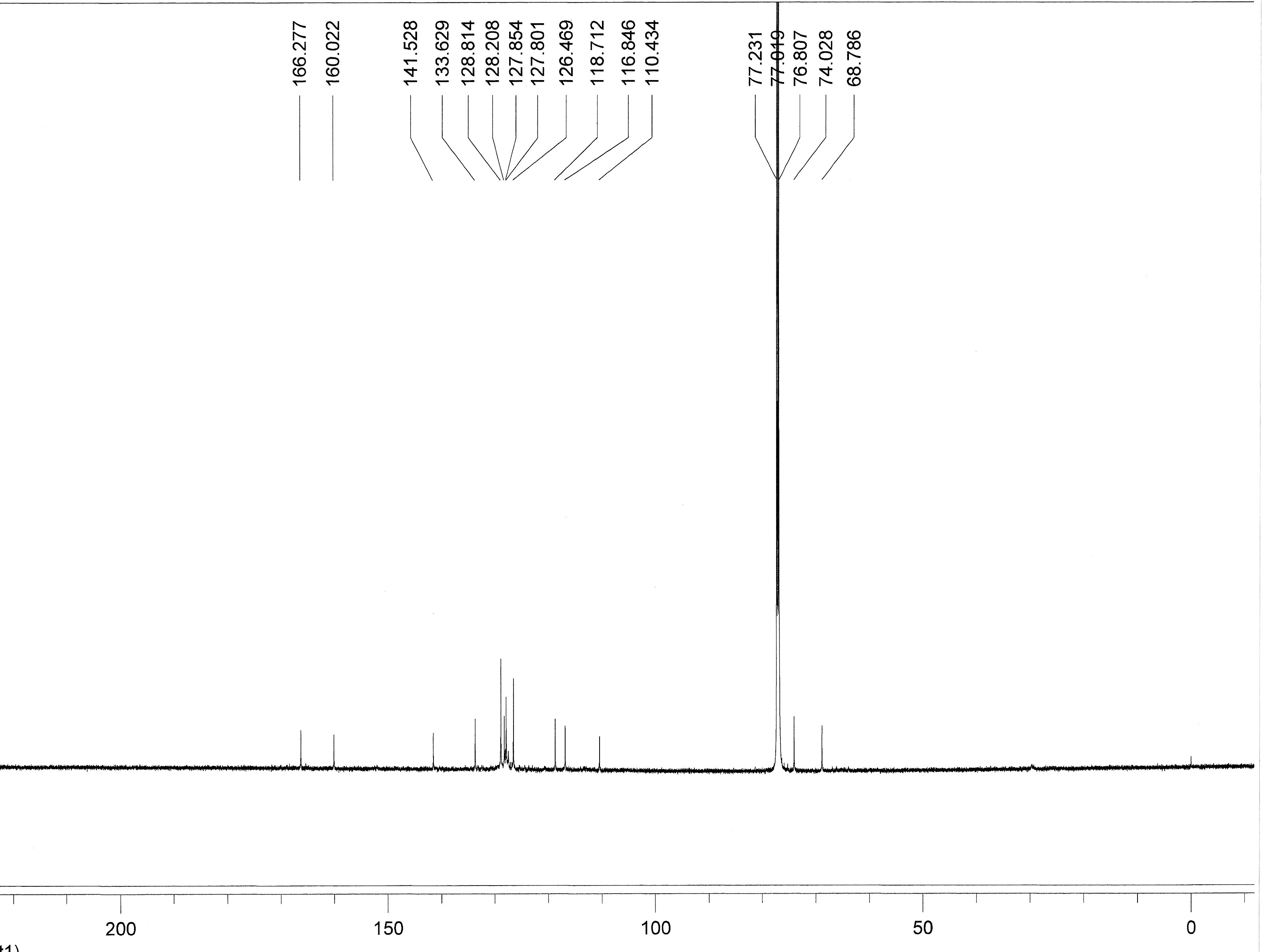


**Complex 4**

**
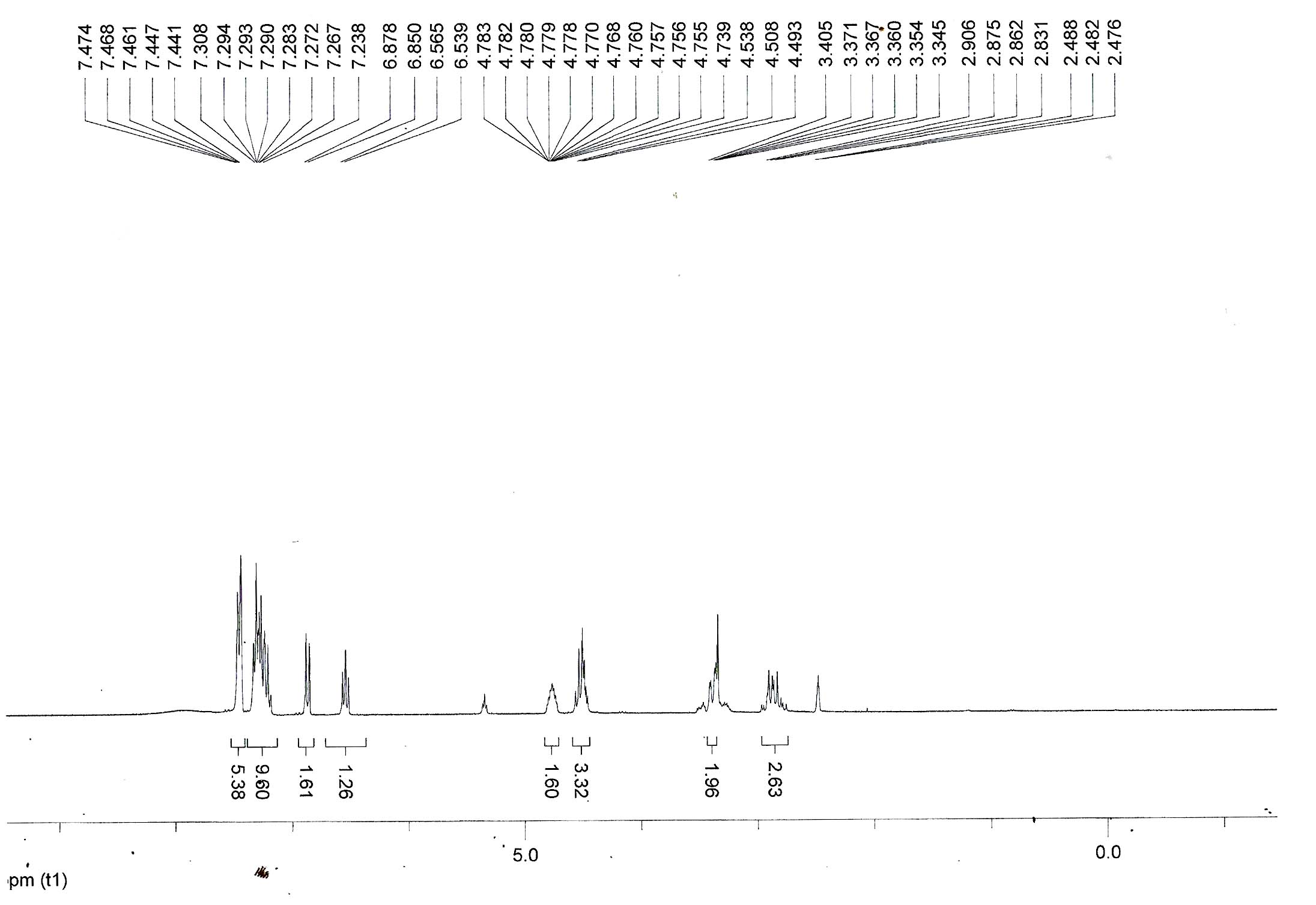
**

**
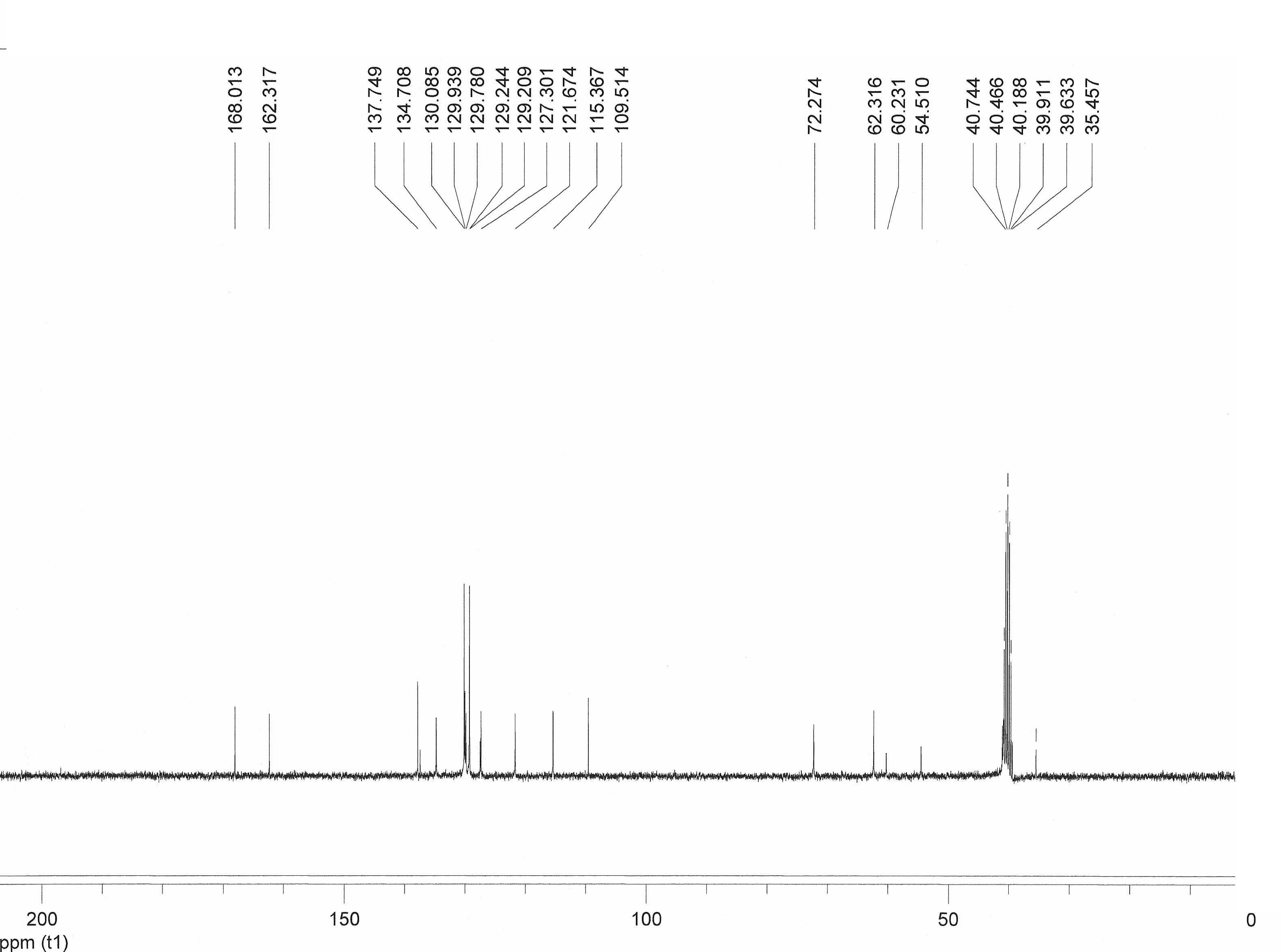
**

**Complex 5**

**
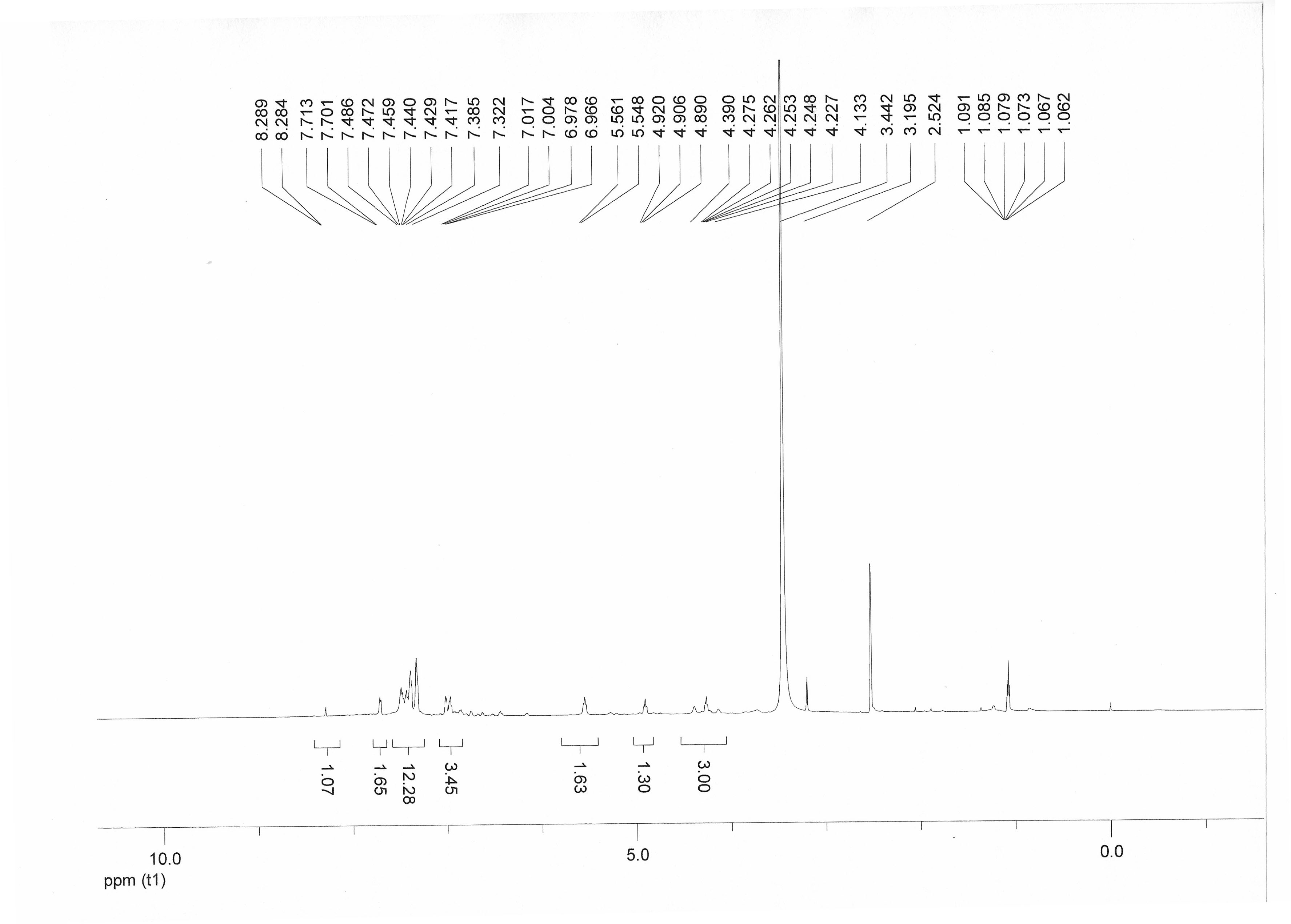
**

**
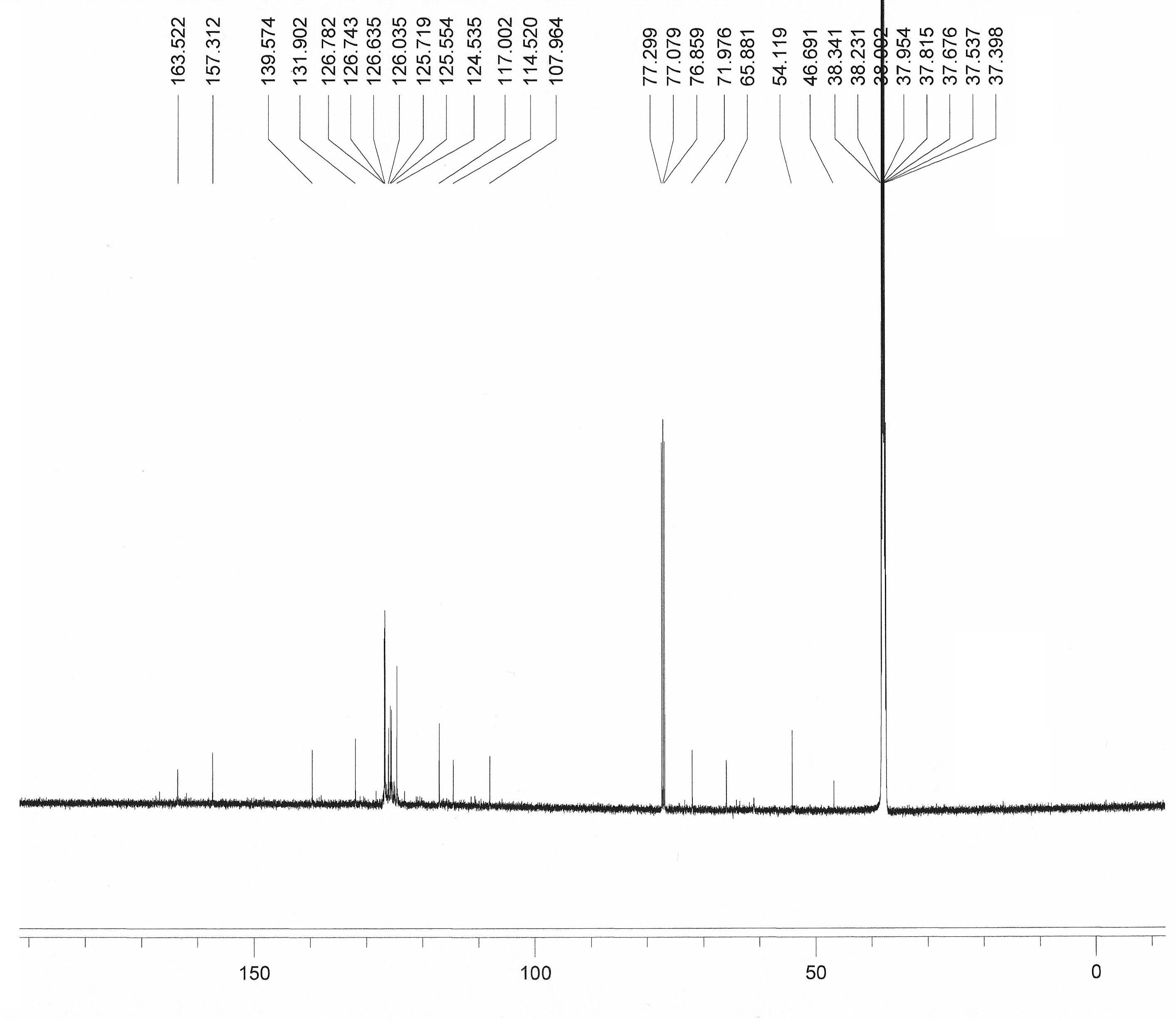
**

**Complex 6**


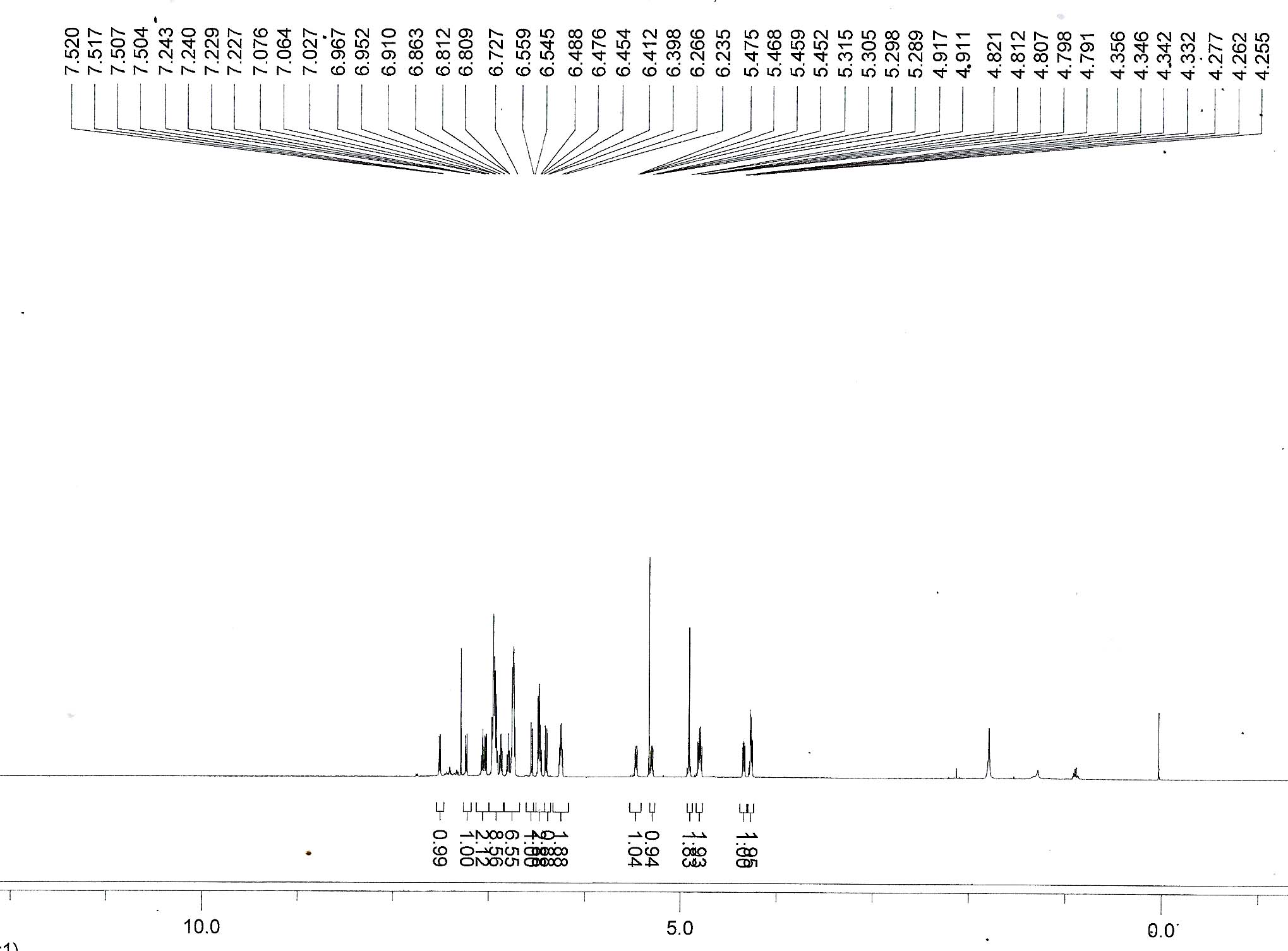


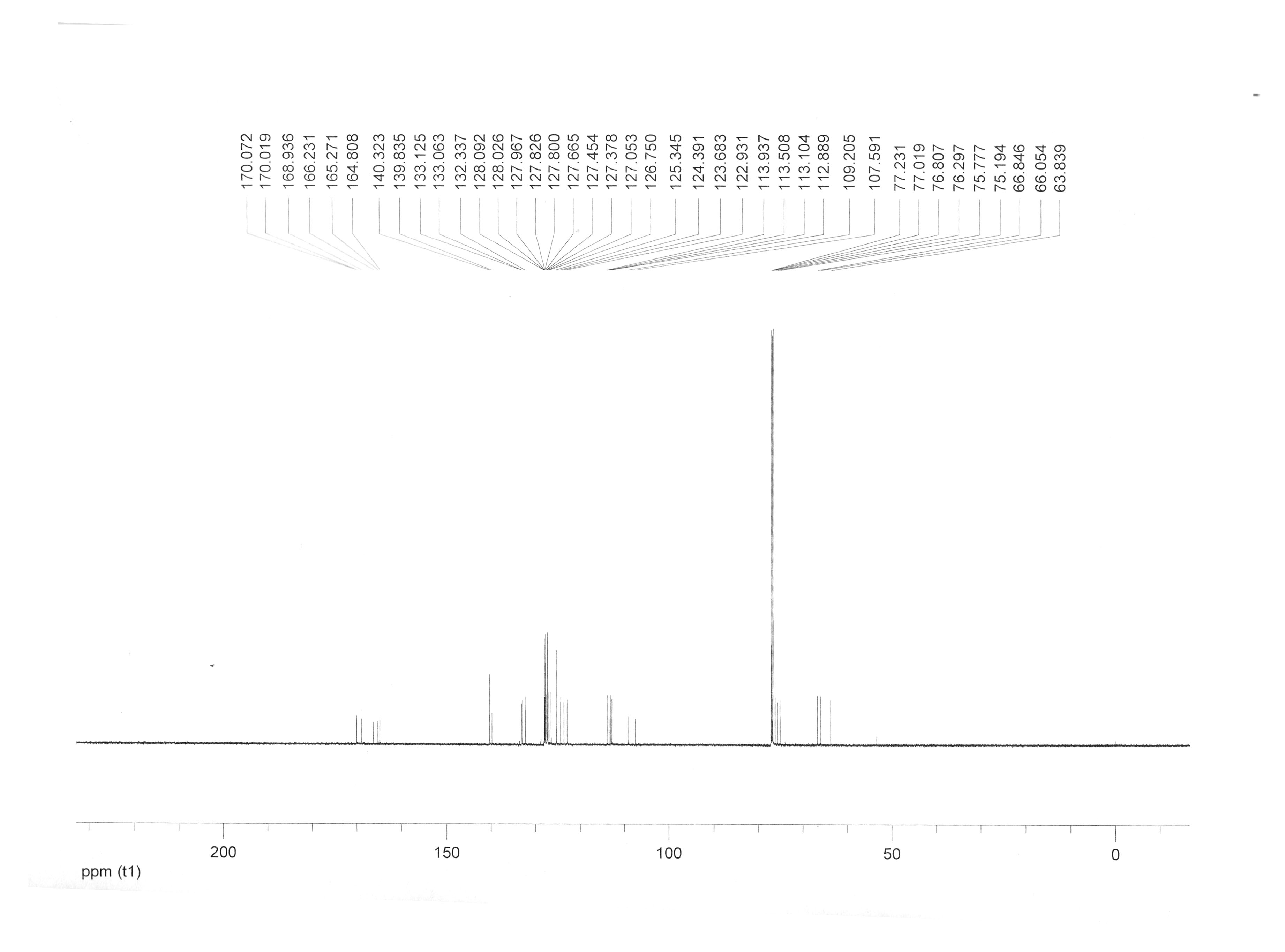


**Complex 7**


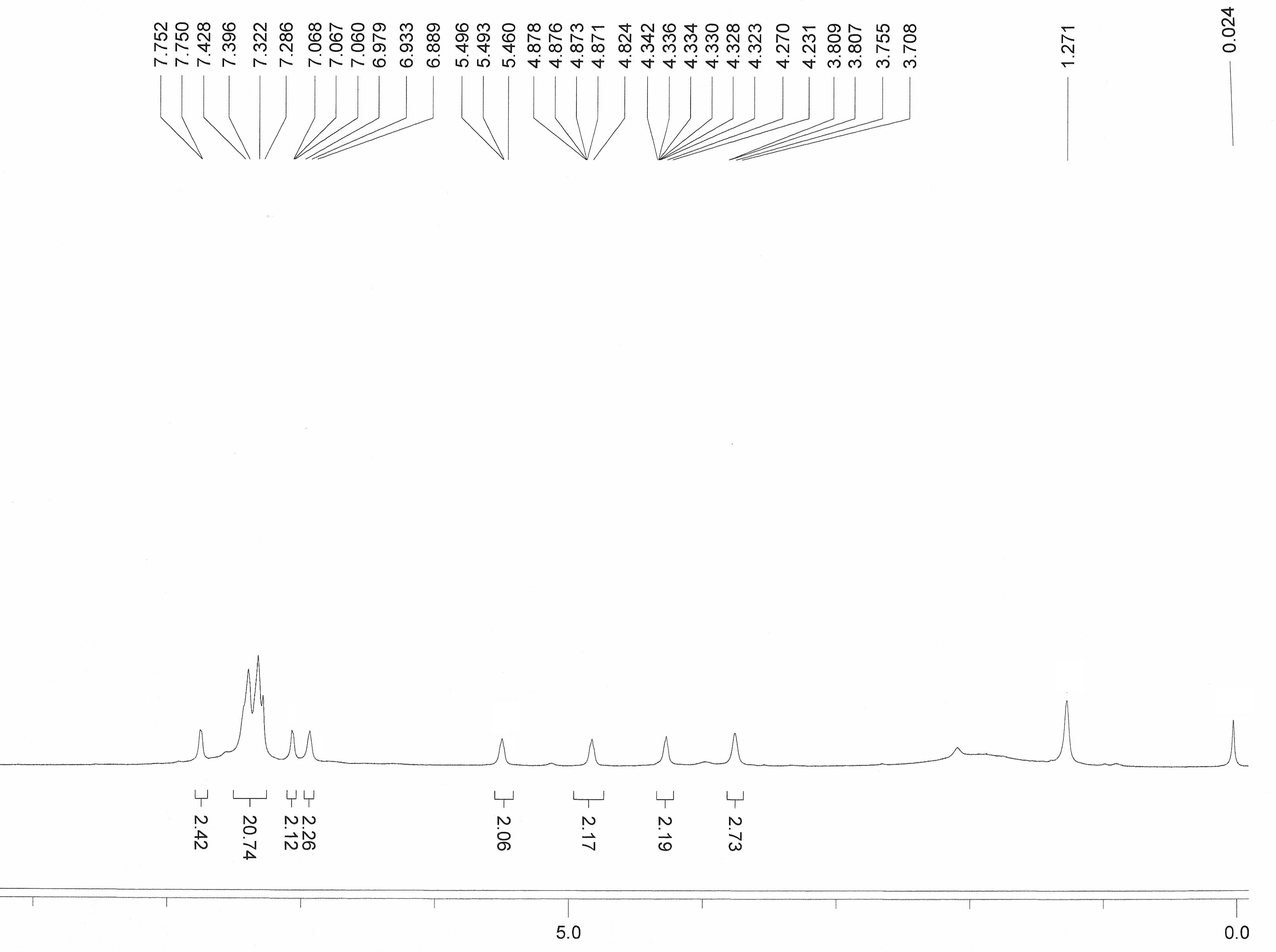


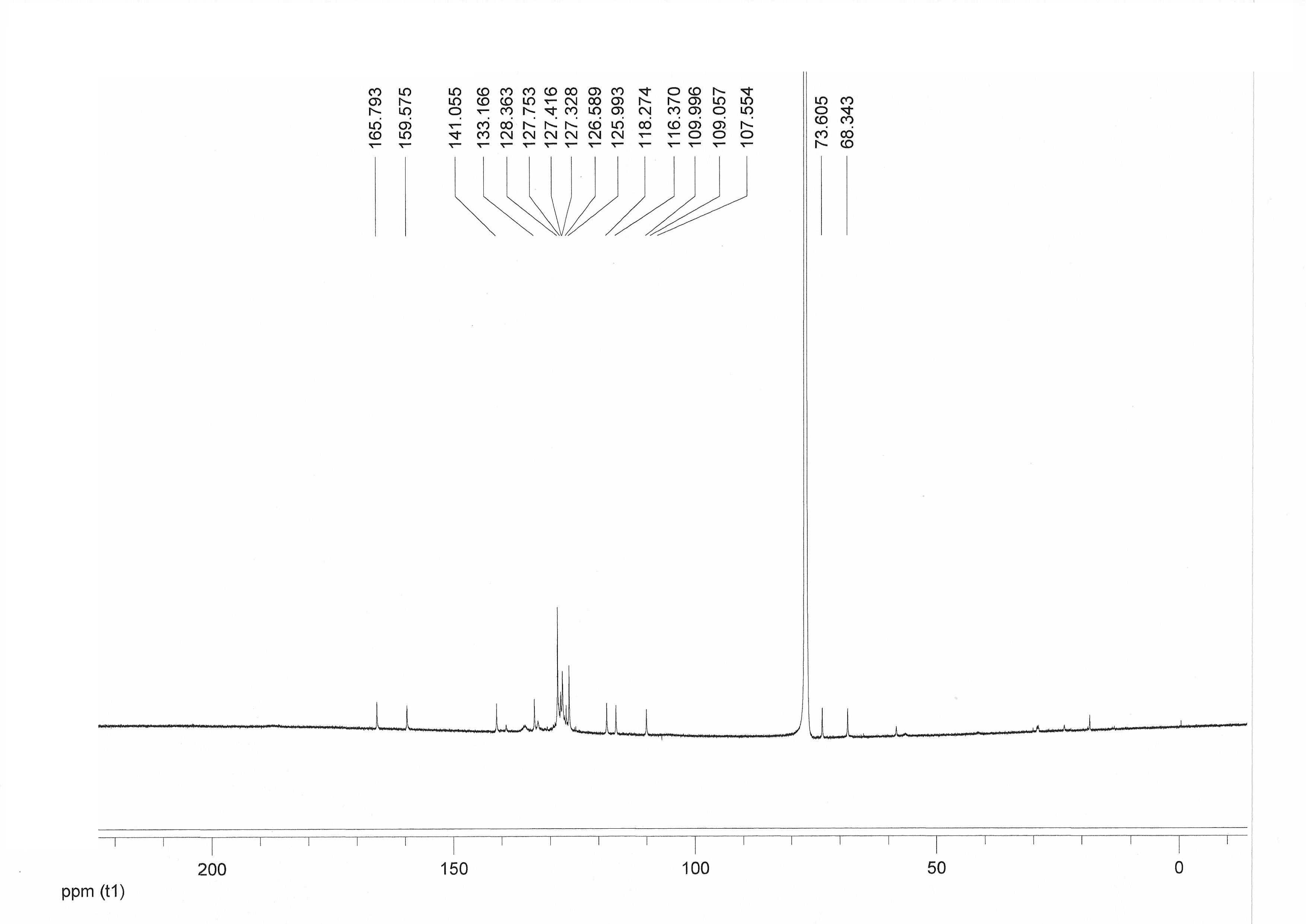


**Complex 8**


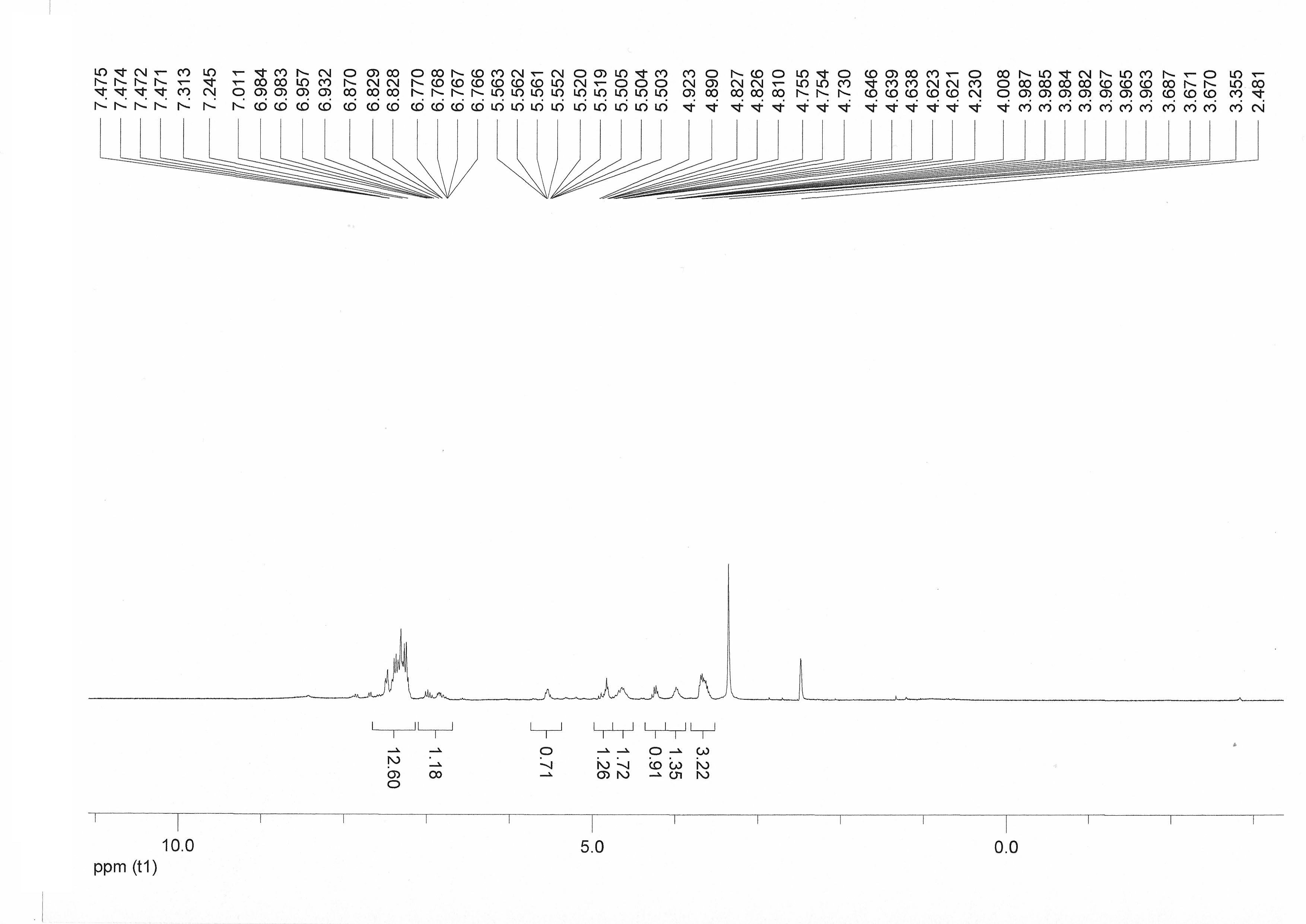


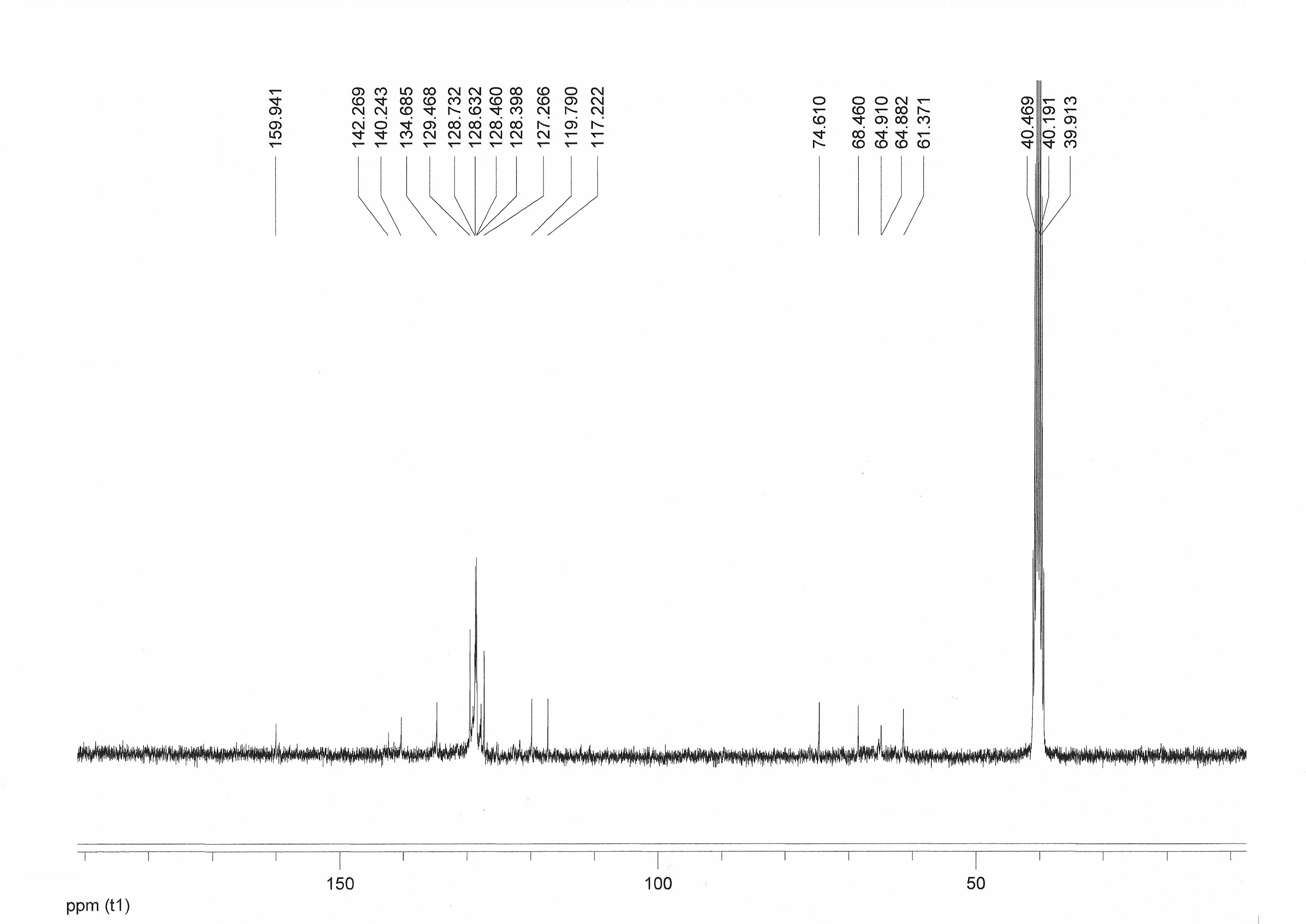

Supplement: Supplementary file 3 — Additional file 3. NMR spectra of complexes 1–8. [file 13065_2019_565_MOESM3_ESM.doc]
